# Regioselectivity in Reactions between Bis(2-benzothiazolyl)ketone and Vinyl Grignard Reagents: *C*- versus *O*-alkylation—Part III †

**DOI:** 10.3390/molecules23010171

**Published:** 2018-01-15

**Authors:** Carla Boga, Silvia Bordoni, Lucia Casarin, Gabriele Micheletti, Magda Monari

**Affiliations:** 1 Department of Industrial Chemistry ‘Toso Montanari’, Alma Mater Studiorum–Università di Bologna Viale Del Risorgimento 4, 40136 Bologna, Italy; silvia.bordoni@unibo.it (S.B.); luciacasarin2000@yahoo.it (L.C.); 2 Department of Chemistry ‘Giacomo Ciamician’, Alma Mater Studiorum–Università di Bologna Via Selmi 2, 40126 Bologna, Italy; magda.monari@unibo.it

**Keywords:** bis(2-benzothiazolyl)ketone, Grignard reagents, vinylmagnesium bromide, *C*-alkylation, *O*-alkylation

## Abstract

The reaction between bis(2-benzothiazolyl)ketone and vinyl Grignard reagents bearing different substituents on the vinyl moiety gave the product derived from attack on the carbonylic carbon- and/or oxygen-atom. The regioselectivity of the attack depends on the kind of substituents bound to the vinylic carbon atoms and on their relative position. The reaction between vinylmagnesium bromide and 2-methyl-1-propenylmagnesium bromide was carried out under different experimental conditions and in the presence of radical scavengers. The results indicate a plausible mechanistic pathway involving radical intermediates in the case of *O*-alkylation, but a polar ones in the case of classic *C*-alkylation. This agrees with our previous reports indicating a key role played by the delocalization ability of the substituents bound to the carbonyl group in driving the regioselectivity of the vinylmagnesium bromide attack towards *O*-alkylation. Further support of this was obtained by diffractometric analysis of four distinct bis(heteroaryl)ketones.

## 1. Introduction

**Scheme 1:**
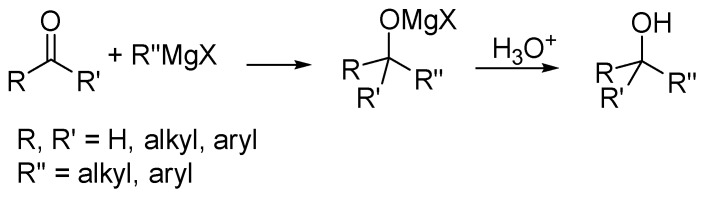
Classic pathway of the addition of Grignard reagents to carbonyl compounds.

Traditionally, Grignard reagents [1] are considered as potential anions able to easily react to hetero double bonds. The most important reaction is the nucleophilic 1,2-addition to the carbonylic carbon atom that gives access to a plethora of alcohol derivatives (Scheme 1).

**Scheme 2:**
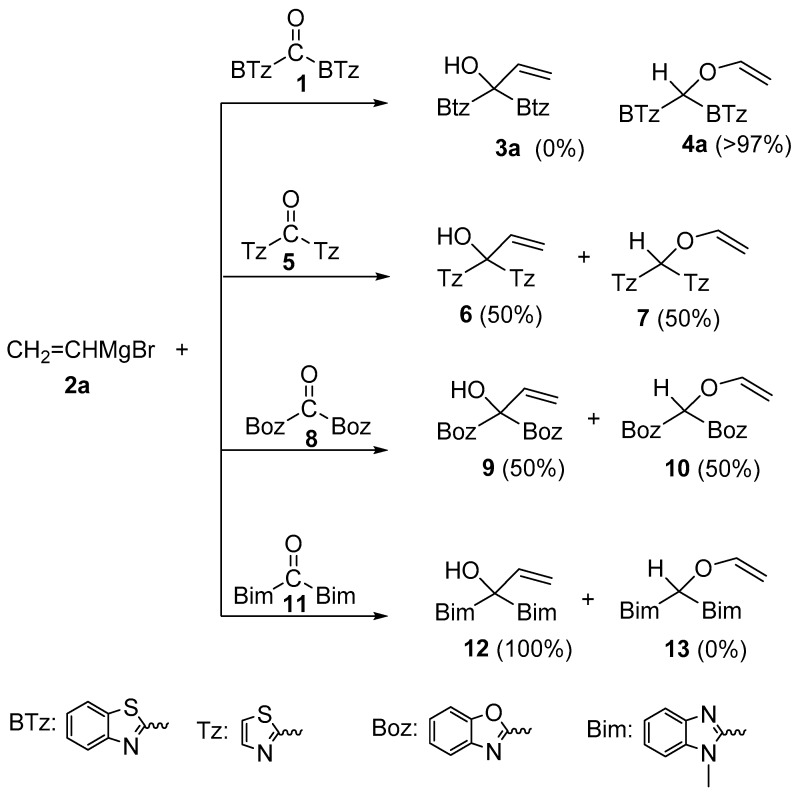
*C*- and *O*-alkylation reactions between vinylmagnesium bromide and several azaheteroaryl ketones.

Depending on the structure of Grignard reagents, substrates or the nature of solvents, alternative reactions can take place [2]. For example, benzophenone reacts with cyclohexyl or isobutyl Grignard reagents forming alcohols [3] or bimolecular reduction to pinacol by using neopentylmagnesium halides; both the classic 1,2-addition and the conjugate addition on the phenyl ring can occur with *tert*-butyl Grignard reagents. *O*-alkylation by Grignard reagents [2] to carbonylic oxygen atom is rarely observed, although it occurred to a minor extent in the case of substrates such as orthoquinones [4], orthoquinolacetates [5,6,7], 9,10-phenanthraquinone [8], and benzil [9]. In our previous studies, we found unexpected reactivity of vinylmagnesium bromide (Scheme 2) and *iso*-propenylmagnesium bromide with di(1,3-benzothiazol-2-yl)ketone (**1**) which gave an exclusive attack to the oxygen carbonyl atom [10]. By reacting **1** and 2-methyl-1-propenylmagnesium bromide, mixtures of *C*- and *O*-alkylation species were obtained in almost equimolar amount. Conversely, alkyl, allyl, or alkynyl Grignard reagents gave exclusively the expected carbinol [11]. *O*-alkylation and concomitant *C*-alkylation [12] occurred with azaheteroaryl ketones such as di(thiazol-2-yl)ketone (**5**) or di(1,3-benzoxazol-2-yl)ketone (**8**). Conversely, di-[*N*-methyl-1,3-benzimidazol-2-yl]ketone (**11**) gave the carbinol as unique compound (see Scheme 2).

More recently, we discovered that phenyl lithium and phenyl Grignard reagents also produced a mixture of *C*- and *O*-alkylation species; the relative ratio is dependent on the substituent on the phenyl ring [13].

Based on the above considerations, to verify the influence of the geometry and the nature of substituents bound to vinyl moiety to *C*- versus *O*-alkylation selectivity, we planned to run reactions between **1** and a series of 1-alkenylmagnesium reagents. Further, we designed an investigation of the crystal structure of the aza-heteroaryl ketones reported in Scheme 2, in order to try to rationalize their different behaviour when they react with vinylmagnesium bromide.

## 2. Results and Discussion

For the sake of simplicity, this part was divided in sub-headings, examining separately the factors that might direct the regioselectivity of the vinyl Grignard reagent attack towards the carbonylic oxygen- or carbon-atom.

### 2.1. Influence of the Stereochemistry of Methyl Substituents on Vinyl Moiety

**Scheme 3:**
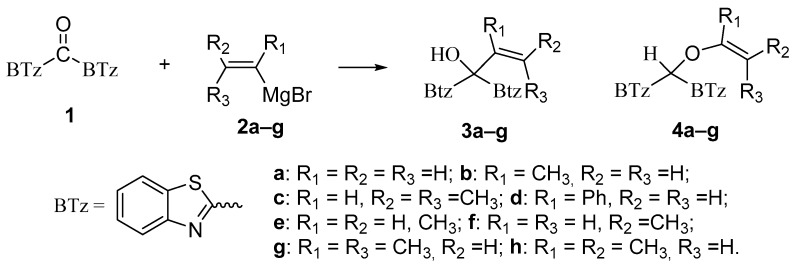
Reactions between **1** and different vinylmagnesium bromides.

The reaction between di(1,3-benzothiazol-2-yl)ketone (**1**) and vinyl magnesium reagents bearing different substituents on the vinyl moiety (Scheme 3) was carried out at −70 °C by adding the Grignard reagent to a solution of ketone in anhydrous tetrahydrofuran. Grignard reagents **2a**–**c** were commercially available whereas **2d**–**h** were synthesized from the suitable vinylbromide and magnesium turnings and titrated prior the use. After 15 min from the addition of the Grignard reagent, the reaction was quenched with water and, after fast work-up (see experimental) the reaction mixture was analysed by ^1^H-NMR spectroscopy and relative molar *C*/*O*-alkylation ratio was inferred from the spectrum. The procedure has been developed since it is known that the *O*-alkylation species can easily undergo air-oxidation of benzylic CH group forming a hemiketal which decomposes to **1** and vinyl alcohol that immediately collapse to the aldehyde tautomeric form. A similar behaviour was also observed for bis(2-benzothiazolyl)methane that spontaneously undergoes oxidation to **1** [14]. Every reaction between **1** and the selected vinyl Grignard was repeated three times and the ratio reported in Table 1 is the average of the three measurements. Compounds **4a**–**g** resulted to be stable enough to be purified by column chromatography on silica gel and stored for at least two months at −18 °C. However, it decomposes as above indicated within 2 days when dissolved in deuterochloroform solvent.

Results obtained are gathered in Table 1. For the sake of comparison, previous data [10] obtained by reacting **1** with vinylmagnesium bromides **2a**–**c** are also reported.

#### 2.1.1. Methyl Substituents in β-Position

The regioselectivity of the reaction between **1** and vinylmagnesium reagents bearing one methyl group in β-position with respect to the C–Mg bond resulted strongly influenced by the stereogeometry and by the electronic feature of the substituent which both affect the alkene stability. In the case of the Grignard Z-isomer (compound **2e**, Table 1, entry 5) the carbinol **3e** and the vinyl ether **4e** were formed in 70/30 relative molar ratio whereas almost complete *O*-alkylation was observed with the *E*-isomer (compound **2f**, Table 1, entry 6).

When the β-dimethyl substituted Grignard reagent **2c** was used (Table 1, entry 3) a 57/43 molar ratio of the *C*/*O*-alkylated products was obtained thus suggesting the prevalence of the e steric hindrance effect (compare entries 3, 5, and 6 in Table 1). This was further confirmed by the experiment with **2g** which gave 90:10 *C*-alkylation:*O*-alkylation ratio indicating that the effect due to the β-substituent prevails over the α-substituent (compare entry 7 with entry 2 in Table 1). To further confirm the influence of the stability of the alkene (mainly due to the substituent electronic effect) and of his geometry in driving the regioselectivity of the attack to the carbonyl group of **1** we attempted to prepare compound **2h** from which we expected to recover almost exclusively the *O*-alkylation product **4h**. Unfortunately, presence of the latter was not detected but the two carbinols **3g** and **3h** in 95:5 relative molar ratio were recovered ascribed to the relevant isomerization occurred during the preparation of the organometallic reagent [15].

#### 2.1.2. Effect of the Substituent in α-Position

Concerning the influence of the substituent in α-position with respect to Mg atom of vinyl Grignard reagents which rules the regioselectivity of the attack towards *C*- or *O*-alkylation, we compared the results reported in entries 1, 2, and 4 of Table 1. Vinylmagnesium bromide (**2a**) and 1-propenylmagnesium bromide (**2b**) reacted with **1** giving exclusively *O*-alkylation, whereas 1-phenylmagnesium bromide gave the carbinol **3d** and the vinyl ether **4d** in 7/3 molar ratio. These findings might be explained by invoking competition between the *C*- and the *O*-attack reactions. From the literature survey about effect of α-substituents to form and stabilize vinyl radicals from the corresponding bromides we found [16] that the configuration of vinyl radicals depends on the nature of the α-substituent. Vinyl radicals with an α-hydrogen, -alkyl, -alkoxy, or-halogen substituent have a bent structure [17,18,19,20] while the cyano, carboxyl, phenyl, and alkenyl group are π-delocalized and thought able to impose linearity on the radical center to effect maximum resonance stabilization, as supported by ESR data [21,22]. Thus, the α-(phenyl)vinyl radical results more stabilized than the α-methyl and vinyl analogues and this might explain the difference found in the behavior with **1**. In other words, the occurrence of both the *O*- and the *C*-alkylation with **2d**, and only the *O*-alkylation with **2a** and **2b** might be explained by the fact that the vinyl radical from **2a** and **2b** would be less stabilized and then more reactive than that derived from **2d** allowing competition between *O*- and *C*-alkylation.

This is in line with the common opinion that anomalous behaviour of Grignard reagents can be explained by invoking a single electron transfer (SET) mechanism [23,24]. It has been reported [2] that the C–Mg bond dissociation energy is well related to the oxidation potential values of different Grignard reagents (the C–Mg dissociation energy of 23 Kcal/mol corresponds an oxidation potential difference of 1.00 Volt [2]) and the vinyl- and aryl-Grignard reagents possess the strongest among the C–Mg dissociation energies [2]. Few cases of *O*-alkylation on alpha-dicarbonyl substrates have been reported, but the reaction occurs with vinyl- and phenyl-Grignard reagents only with 9,10-phenanthraquinone [8]. The authors hypothesized that the nature of C-Mg bond is an important requisite to determine the fashion-mode of addition.

#### 2.1.3. Effect of the Substituent in α-Position

**Scheme 4:**
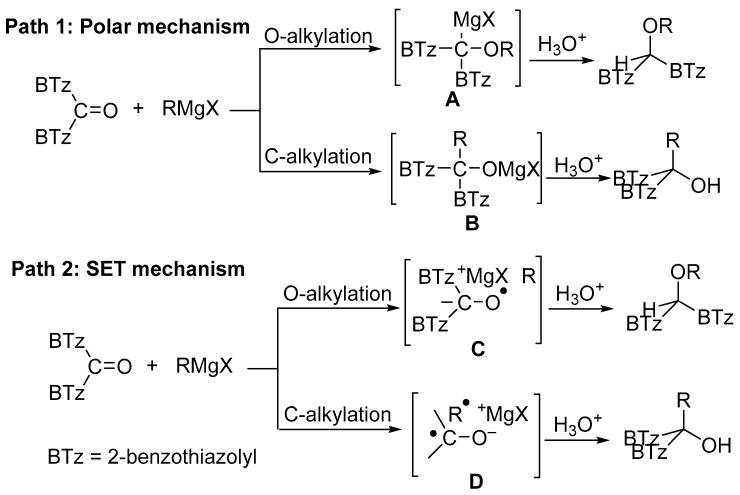
Possible reaction pathways of the currently considered Grignard addition.

It globally emerges from the reported data that the regioselectivity of the vinyl Grignard addition to **1** cannot be exclusively attributed to both steric and electronic features of the vinyl moiety substituents but also to mechanistic path effects. In the context, distinct intermediate species can be involved in *O*- and *C*-alkylation deriving by single electron transfer (SET) or polar mechanism. Some of them are drawn in Scheme 4.

To shed light on the mechanistic aspects of the reaction we planned to run some experiments and results obtained are reported in Table 2. In particular, compound **1** was reacted with vinylmagnesium bromide (**2a**), and 2-methyl-1-propenylmagnesium bromide (**2c**) under different experimental conditions. We selected to run experiments by using these latter since they are representative of limit cases and exclude isomerization, thus avoiding further complications. Indeed, **2a** gave only *O*-alkylation, while both *C*- and *O*-alkylation occurred with **2c**.

**Scheme 5:**
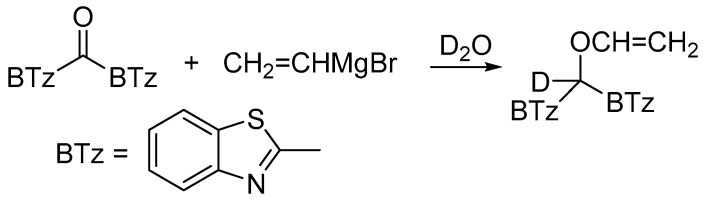
Formation of **4a** deuterated on the benzylic position upon quenching with D_2_O of the reaction between **1** and **2a**.

From data reported in Table 2 emerges that the conversion of **1** decreased in the presence of 2,2,6,6-tetramethylpiperidinoxyl (TEMPO) as radical scavenger (comparison between entries 1 and 2); the recovery of the *O*-alkylation product even in the presence of the scavenger might be due to the coupling rate of the vinyl radical with **1**, likely faster than the diffusion rate of the scavenger within the solvent cage. When the radical trapper was present in half equivalent amount with respect to the reactant amount, the reaction between **1** and **2c** (Table 2, entries 3–5) gives **3c**:**4c** = 2:1 in minor conversion, whereas only the carbinol **3c** was detected when TEMPO was added in equimolar amount. These results can be considered an indication that the *O*-alkylation involves the intervention of **C**-like radical species (Scheme 4). The recovery of the totally deuterated species **4a** from the crude reaction mixture from **1** and **2a** upon quenching with D_2_O [12] supported the above speculation (Scheme 5).

Finally, we made a further experiment aiming to support the hypothesis that the reaction between **1** and vinyl Grignard reagents gives *C*-alkylation through a polar mechanism while *O*-alkylation proceeds involving radical species. Ashby reported fundamental studies on the mechanism of reaction of Grignard reagents with ketones. In particular, he reported experiments in which propenylmagnesium reagents having different *cis*/*trans* ratios were reacted with benzophenone excess [25,26]. The c*is*-propenylmagnesium bromide was selected as vinyl Grignard probe. If a free radical would be formed during the reaction course, the *cis*-propenyl radical would isomerize to the thermodynamically stable *trans*-propenyl radical, resulting in a product with *trans* stereochemistry. The result [25] did not indicate isomerization of the propenyl group as evidenced by the fact that the starting Grignard reagent *cis*/*trans* ratio was exactly reflected in the reaction products. Thus, the retention of configuration of the probe was considered an indication that the reaction is not proceeding via an SET mechanism.

In line with these considerations, we prepared a THF solution of propenylmagnesium bromide by reacting *cis*-propenylbromide with magnesium turnings dividing it in two parts: one to be reacted with benzophenone while the other with bis(2-benzothiazolyl)ketone (**1**). The ^1^H-NMR analysis of the reaction mixture with benzophenone showed two carbinol signals derived from the addition of *cis*- and *trans*-propenylmagnesium bromide to benzophenone. In agreement with the result reported in Table 1, entry 5, the *cis*:*trans* ratio was 1:2, indicating that the isomerization occurs in Grignard formation but not during the addition to the ketone.

In the case of reaction with **1**, the ^1^H-NMR spectrum exhibited signals ascribed to carbinols **3e** and **3f** in relative 1:2 molar ratio and of *O*-alkylated products **4e** and **4f**. These findings indicate that carbinols derive from a polar mechanism, as in the case of the reaction with benzophenone. The detection of vinyl ethers suggests the occurrence of a radical mechanism when bis(2-benzothiazolyl)ketone was used as substrate.

### 2.2. Influence of the Solvent and of the Metal Bound to Vinyl Group

It has been reported [9] that the reaction between benzil and phenylmagnesium bromide in THF gives *O*-alkylation (25% yield), whereas this reaction occurred in negligible amount in Et_2_O. Therefore, addition of **2a** to a solution of **1** in diethyl ether afforded the *C*- and *O*-alkylation in equimolar extent, although the reaction is slow due to the low solubility of **1** in Et_2_O confirming a strong influence of the solvent polarity to the regioselectivity.

**Scheme 6:**
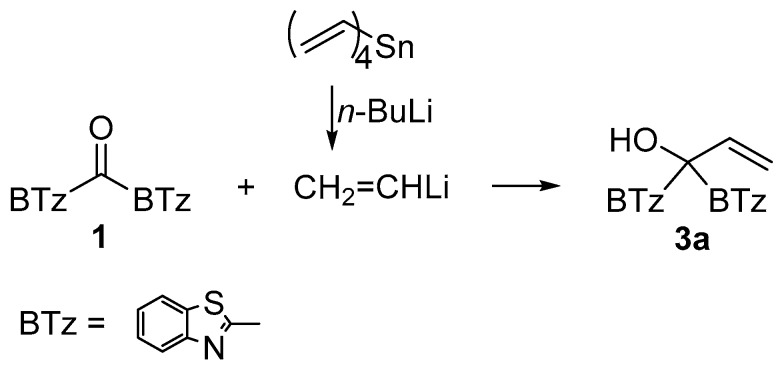
Synthesis of carbinol **3a**.

To verify the role of the alkylating agent on the regioselectivity we run the reaction between **1** and vinyl lithium, prepared in situ from tetravinylstannane and *n*-butyllithium. After purification by silica gel chromatography the carbinol **3a** in 25% yield was recovered and no *O*-alkylated product was detected. This finding can be considered of synthetic interest since the vinyllithium offers an alternative synthetic path to obtain the *C*-alkylation product **3a** (Scheme 6).

### 2.3. X-ray Diffraction Analysis of Azaheteroaryl Ketones Showing Different C-/O-Alkylation Regioselectivity towards Vinylmagnesium Bromide

It has been reported [10,12] that the reaction between **2a** and a series of azaheteroaryl ketones exhibited *O*-vinylation strongly dependent on the groups bound to the carbonyl moiety. It occurred completely only with the ketone 1, bearing two benzothiazolyl substituents while the reaction with bis(2-thiazolyl)ketone (**5**) produced both *O*- and *C*-alkylation products in equimolar amount (Scheme 2) evidencing the importance of the benzocondensation. The presence of a benzene ring fused with the heterocycle might favor the delocalization of a negative charge in the *O*-alkylated C-like intermediate (Scheme 4). Equimolar amount of two distinct *O*- and *C* products were obtained on bis(2-benzoxazolyl)ketone (**8**) substrate. Conversely, in the case of bis(1-methylbenzimidazolyl)ketone (11) the carbinol species derived from the classic Grignard addition was exclusively obtained. These findings suggested us to investigate the structure of ketones **1**, **5**, **8**, and **11** by diffractometric analysis. Suitable crystals for the purpose were obtained from acetone (**1** and **11**), methanol (**8**), or by the vapor diffusion technique (methanol/*n*-hexane) in the case of **6**.

**Figure 1:**
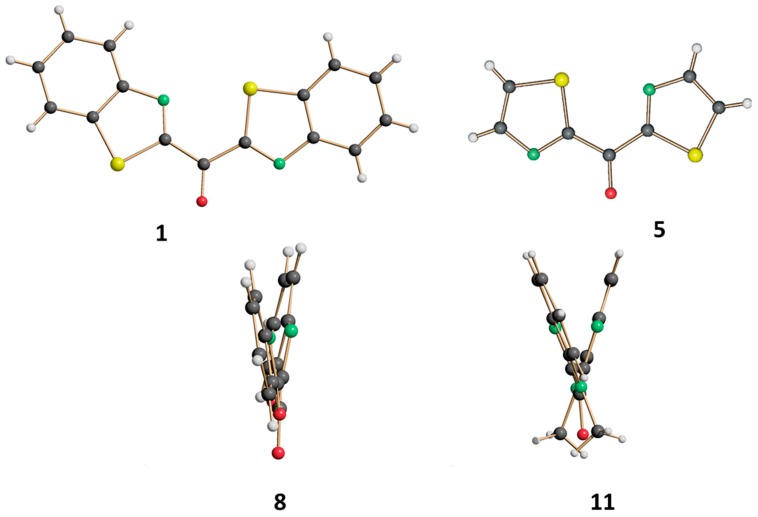
Molecular structures of compounds **1**, **5** (front-view), **8**, **11** (side-view) determined by X-ray studies.

X-ray study showed distinct stereogeometries characterized by different dihedral angles (Table 3) between the planes defined by the two carbonyl substituents, as shown in Figure 1.

In compounds **1** and **5** in which the ketone group is bound to two benzothiazolyl and thiazolyl rings, respectively, the dihedral angles are 12.9(1)° and 5.0(1)° [8.3(1)°, for the second molecule in **5**]. At a difference with the previously observed values, in **8** the dihedral angles formed by the benzoxazolyl rings bound to the carbonyl group are slightly wider being 22.76(7)° whereas the largest angle is found between the methylbenzoimidazolyl rings in 11 [39.76(7)°]. This latter result is also in agreement with the analogous dihedral angle between the methylimidazole rings in the bis(1-methyl-2-imidazolyl)ketone that is even larger being 49.4(1)° [27].

Although we are aware that conformations in the solid state can differ from the ones in solution, by roughly comparing the structure geometry of the benzocondensed azaheteroaryl ketones **1**, **8**, and **11**, it emerges that the attack at the oxygen atom may be sterically impeded by the presence of two *N*-methyl groups in the case of **11**.

A further possible explanation about the difference between relative *C*-and *O*-alkylation ratio passing from **1** to **8** until **11** might reside in the distinct ability to delocalize a negative charge on the benzylic carbon atom. In the case of **11** the presence of nitrogen atoms makes the imidazole ring more aromatic thus less prone to delocalize the charge within the ring; when the nitrogen is replaced by the oxygen such as in **8** this feature might be less important, whereas the intervention of the sulfur d-orbitals in the case of **1** might likely favor the delocalization charge.

## 3. Materials and Methods

### 3.1. General Methods

The ^1^H spectra were recorded with a Varian Gemini 300 (Varian, Palo Alto, CA, USA) spectrometer operating at 300 MHz for ^1^H-NMR and 75.56 MHz for ^13^C-NMR. Signal multiplicities were established by Distortionless Enhanced by Polarization Transfer (DEPT) experiments. Chemical shifts were measured in δ (ppm) with reference to the solvent (δ = 7.26 ppm and 77.00 ppm for CDCl_3_, for ^1^H-, and ^13^C-NMR, respectively). *J* values are given in Hz. MS analyses were performed with a gaschromatograph Agilent 6890 equipped with a (5%-phenyl)methyl-polysiloxane column (30 m length, 0.250 mm i.d., 0.25 µm thickness) interfaced to a quadrupole mass detector Agilent 5973N. Mass and HR-MS spectra were recorded on a VG 7070E instrument (VG Instruments, Inc.， Stamford, CT, USA) at an ionization voltage of 70 eV in the EI mode. Electron spray ionization mass spectra (ESI-MS) were recorded with a WATERS 2Q 4000 instrument (Waters Corporation, Milford, MA, USA). IR spectra were recorded on a Perkin–Elmer model 1600 FT-IR spectrophotometer (Perkin Elmer, Waltham, MA, USA). Compounds **3a**–**h** showed characteristic IR signals at 3650–3300 cm^−1^ (OH) and **4a**–**g** (oils) at 1050–1300 cm^−1^ (O–C). Melting points were measured with a Büchi 535 apparatus and were not corrected. Chromatographic purifications (FC) were carried out on glass columns packed with silica gel (Merck grade 9385, 230–400 mesh particle size, 60 Å pore size) at medium pressure. Thin layer chromatography (TLC) was performed on silica gel 60 F254 coated aluminum foils (Fluka Chemie GmbH, Buchs, Switzerland). The solvents and reagents used are commercially available (Sigma-Aldrich (Milan, Italy) or Fluka Chemie GmbH, Buchs, Switzerland). THF and diethyl ether were distilled from sodium/benzophenone ketyl. Air- and moisture-sensitive solutions and reagents were handled in dried apparatus under an atmosphere of dry argon using standard Schlenk-type techniques. Compounds **1**, **5**, **8**, and **11** are synthesized as previously reported [14] and their characterization data agreed with those reported. The Grignard reagents, if not commercially available, were prepared from the vinyl bromide precursor and their solution titrated immediately prior to use according to standard procedure [28].

### 3.2. Synthesis of 1,1-Di(1,3-benzothiazol-2-yl)-2-propen-1-ol (***3a***)

In a three necked round bottom flask equipped with two drip funnels and with a neck settled with a sintered glass filter shut with a Teflon valve, under argon atmosphere and magnetic stirring, a solution of tetravinyltin (97% purity grade, 0.36 mL, 1.92 mmol) in anhydrous *n*-pentane (120 mL) was introduced. A solution of *n*-butyllithium 2.5 M in *n*-hexane (1.8 mL) was added dropwise and after 30 min the white solid obtained was separated by filtration from the liquid through the filter and washed with 2.0 mL of *n*-pentane. The solid remained within the flask was dissolved in anhydrous THF (5.0 mL) and the apparatus was cooled at −70 °C through immersion in a liquid nitrogen/acetone bath. After about 5 min, a solution of compound **1** (0.065, 0.22 mmol) in anhydrous THF (10 mL) was added dropwise. Immediately the pale yellow solution became red. The reaction course was monitored through TLC and after 2 h 10 mL of water was added; the crude was extracted with diethyl ether and the organic layer dried over anhydrous Na_2_SO_4_. Compound **3a** was recovered in 20% yield after purification by column chromatography on silica gel (eluent: light petroleum/diethyl ether 75/25).

*1,1-Di(1,3-benzothiazol-2-yl)-2-propen-1-ol* (**3a**): ^1^H-NMR (CDCl_3_, 300 MHz, 25 °C): δ (ppm): 8.08–8.02 (m, 2H), 7.90–7.85 (m, 2H), 7.52–7.44 (m, 2H), 7.42–7.35 (m, 2H), 6.77 (dd, 1H, *J* = 17.0 Hz, *J* = 10.4 Hz), 5.77 (d, 1H, *J* = 17.0 Hz), 5.63 (bs, 1H, OH), 5.42 (d, 1H, *J* = 10.4 Hz); ^13^C-NMR (CDCl_3_: 75.56 MHz, 25 °C): δ (ppm): 173.7, 152.4, 139.2, 135.9, 126.1, 125.4, 123.3, 121.9, 116.1, 78.9; MS (*m*/*z*): 324 (M^+^, 76), 307 (25), 296 (25), 268 (27), 190 (38), 162 (100), 136 (41). HREIMS: *m*/*z* 324.0395 (calculated for C_17_H_12_N_2_OS_2_, 324.0391).

### 3.3. Reactions between ***1*** and Vinylmagnesium Bromide Derivatives (***2a***–***h***)—General Procedure

Vinylmagnesiumbromide derivative (0.41 mmol) in THF (3.0 mL) was added dropwise to a stirred solution of carbonyl compound (0.34 mmol) in THF (3.0 mL), cooled at −70 °C. After about 20 min. the reaction mixture was quenched with saturated aqueous (NH_4_)_2_SO_4_, stirred while being warmed to room temperature, and extracted with diethyl ether. The organic layers were washed with “brine”, dried over anhydrous Na_2_SO_4_, and concentrated “in vacuo” without warming. The time employed for this workup was about 15 min. The residue was analyzed by ^1^H-NMR and then the products were isolated by FC and fully characterized. The vinyl ethers, stored at −18 °C, are stable for several months. Reactions of **1** with **2a**–**c** and related products have been reported in the literature [10]. Characterization data for new compounds are reported below and the relative **3**:**4** ratio is reported in Table 1.

*1,1-Di(1,3-benzothiazol-2-yl)-2-phenyl-2-propen-1-ol* (**3d**): ^1^H-NMR (CDCl_3_, 300 MHz, 25 °C): δ (ppm): 8.03 (d, *J* = 8.0 Hz, 2H), 7.83 (d, *J* = 7.4 Hz, 2H), 7.50–7.30 (m, 6H), 7.22–7.14 (m, 3H), 5.96 (bs, 1H, OH, exchange with D_2_O), 5.56 (s, 1H), 5.47 (s, 1H); ^13^C-NMR (CDCl_3_: 75.56 MHz, 25 °C): δ (ppm): 173.8, 152.2, 150.6, 138.7, 136.1, 128.8, 127.8, 127.6, 126.1, 125.4, 123.4, 121.7, 120.1, 81.3; ESI-MS (*m*/*z*): 401 [M + H]^+^, 423 [M + Na]^+^; HREIMS: *m*/*z* 400.0711 (calculated for C_23_H_16_N_2_OS_2_, 400.0704).

*Z-1,1-Bis(2-benzothiazolyl)-2-buten-1-ol* (**3e**): ^1^H-NMR (CDCl_3_, 300 MHz, 25 °C): δ (ppm): 8.08–7.98 (m, 2H), 7.88–7.82 (m, 2H), 7.50–7.40 (m, 2H), 7.40–7.30 (m, 2H), 6.46 (dq, 1H, *J* = 11.2 Hz, *J* = 1.8 Hz), 6.05–5.90 (m, 1H), 5.73 (bs, 1H, OH), 1.76 (dd, 3H, *J* = 7.3 Hz, *J* = 1.8 Hz); ^13^C-NMR (CDCl_3_: 75.56 MHz, 25 °C): δ (ppm): 175.6, 152.4, 136.0, 132.8, 131.7, 126.1, 125.3, 123.3, 121.8, 78.3, 14.7; MS (*m*/*z*): 338 (M^+^), 321, 297, 281, 176, 136; HREIMS: *m*/*z* 338.0543 (calculated for C_18_H_14_N_2_OS_2_, 338.05475).

*E-1,1-Bis(2-benzothiazolyl)-2-buten-1-ol* (**3f**): ^1^H-NMR (CDCl_3_, 300 MHz, 25 °C): δ (ppm): 8.10–7.95 (m, 2H), 7.90–7.80 (m, 2H), 7.55–7.42 (m, 2H), 7.42–7.35 (m, 2H), 6.40 (dq, *J* = 15.3 Hz, *J* = 1.5 Hz, 1H), 6.22–6.05 (m, 1H), 5.63 (br.s., 1H, OH, exchange with D_2_O), 1.78 (dd, *J* = 6.6 Hz, *J* = 1.5 Hz, 3H); ^13^C-NMR (CDCl_3_: 75.56 MHz, 25 °C): δ (ppm): 174.5, 152.4, 135.9, 132.5, 128.2, 126.1, 125.3, 123.2, 121.8, 78.6, 17.6; MS (*m*/*z*): 338 (M^+^), 321, 297, 281, 176, 136; HREIMS: *m*/*z* 338.0546 (calculated for C_18_H_14_N_2_OS_2_, 338.05475).

*Z-1,1-Bis(2-benzothiazolyl)-2-methyl-2-buten-1-ol* (**3g**): ^1^H-NMR (CDCl_3_, 300 MHz, 25 °C): δ (ppm): 8.05 (d, 2H, *J* = 7.4 Hz), 7.89 (d, 2H, *J* = 8.22 Hz), 7.53–7.35 (m, 4H), 5.81–5.77 (m, 1H), 5.62 (br.s., 1H, OH), 1.85 (s, 3H), 1.47–1.43 (m, 3H); ^13^C-NMR (CDCl_3_: 75.56 MHz, 25 °C): δ (ppm): 175.1, 152.3, 136.6, 136.6, 136.0, 128.6, 126.1, 125.4, 123.4, 121.8, 80.4, 23.1, 15.0; MS (*m*/*z*): 352 (M^+^), 335, 320, 297, 190, 162; HREIMS: *m*/*z* 352.0707 (calculated for C_19_H_16_N_2_OS_2_, 352.0704).

*E-1,1-Bis(2-benzothiazolyl)-2-methyl-2-buten-1-ol* (**3h**): m.p.: 123–124 °C. ^1^H-NMR (CDCl_3_, 300 MHz, 25 °C): δ (ppm): 8.05 (d, 2H, *J* = 8.1 Hz), 7.90 (d, 2H, *J* = 8.4 Hz), 7.50 (td, 2H, *J* = 8.1 Hz, *J* = 1.3 Hz), 7.40 (td, 2H, *J* = 8.4 Hz, *J* = 1.2 Hz), 5.70 (bs, 1H, OH), 5.70–5.55 (m, 1H), 1.80 (s, 3H), 1.70 (d, 3H, *J* = 6.8 Hz); ^13^C-NMR (CDCl_3_: 75.56 MHz, 25 °C): δ (ppm): 174.1, 152.3, 137.8, 136.2, 126.1, 125.4, 125.3, 123.3, 121.8, 82.3, 13.9, 12.8; MS (*m*/*z*): 352 (M^+^), 335, 320, 297, 190, 162; HREIMS: *m*/*z* 352.0708 (calculated for C_19_H_16_N_2_OS_2_, 352.0704).

*2-[1,3-Benzothiazol-2-yl((1-phenylvinyl)oxy)methyl]-1,3-benzothiazole* (**4d**): ^1^H-NMR (CDCl_3_, 300 MHz, 25 °C): δ (ppm): 8.09 (d, 2H, *J* = 8.2 Hz), 7.88 (d, 2H, *J* = 8.0 Hz), 7.80–7.35 (m, 9H), 7.03 (s,1H), 4.91 (d, 1H, *J* = 3.9 Hz), 4.62 (d, 1H, *J* = 4.0 Hz); ^13^C-NMR (CDCl_3_: 75.56 MHz, 25 °C): δ (ppm): 168.1, 157.6, 153.0, 135.2, 129.1, 128.4, 126.2, 125.6 (two signals overlapped), 123.9, 121.8, 87.6, 77.8; MS (*m*/*z*): 400 (M^+^), 399 (M^+^–1), 281; HREIMS: *m*/*z* 400.0709 (calculated for C_23_H_16_N_2_OS_2_, 400.0704).

*Z-Bis-(2-benzothiazolyl)methyl 1-propenyl ether* (**4e**): ^1^H-NMR (CDCl_3_, 300 MHz, 25 °C): δ (ppm): 8.10–8.00 (m, 2H), 7.92–7.80 (m, 2H), 7.52–7.30 (m, 4H), 6.53 (s, 1H), 6.29 (dq, 1H, *J* = 6.1 Hz, *J* = 1.8 Hz), 4.72 (dq, 1H, *J* = 12.8 Hz, *J* = 6.9 Hz), 1.80 (dd, 3H, *J* = 6.9 Hz, *J* = 1.8 Hz); ^13^C-NMR (CDCl_3_: 75.56 MHz, 25 °C): δ (ppm): 168.3, 152.9, 142.8, 135.3, 126.2, 125.6, 123.8, 121.8, 106.3, 80.6, 9.6; MS (*m*/*z*): 400 (M^+^), 338 (M^+^), 281, 162, 134; HREIMS: *m*/*z* 338.0543 (calculated for C_18_H_14_N_2_OS_2_, 338.05475).

*E-Bis-(2-benzothiazolyl)methyl 1-propenyl ether* (**4f**): ^1^H-NMR (CDCl_3_, 300 MHz, 25 °C): δ (ppm): 8.12–8.02 (m, 2H), 7.92–7.82 (m, 2H), 7.52–7.30 (m, 4H), 6.58 (s, 1H), 6.42 (dq, 1H, *J* = 12.4 Hz, *J* = 1.7 Hz), 5.22 (dq, 1H, *J* = 12.4 Hz, *J* = 6.8 Hz), 1.54 (dd, 3H, *J* = 6.8 Hz, *J* = 1.6 Hz); ^13^C-NMR (CDCl_3_: 75.56 MHz, 25 °C): δ (ppm): 168.3, 153.2, 143.7, 135.2, 126.2, 125.6, 123.8, 121.8, 105.2, 79.2, 12.4; MS (*m*/*z*): 338 (M^+^), 281, 162, 134; HREIMS: *m*/*z* 338.0541 (calculated for C_18_H_14_N_2_OS_2_, 338.05475).

*Z-Bis(2-benzothiazolyl)methyl 2-butenyl ether* (**4g**): ^1^H-NMR (CDCl_3_, 300 MHz, 25 °C): δ (ppm): 8.10–8.00 (m, 2H), 7.90–7.80 (m, 2H), 7.50–7.35 (m, 4H), 6.80 (s, 1H), 4.75–4.65 (m, 1H), 1.98–1.94 (m, 3H), 1.78–1.75 (m, 3H); REIMS: *m*/*z* 352.0700 (calculated for C_19_H_16_N_2_OS_2_, 352.0704).

### 3.4. Reactions of Propenylmagnesium Bromide with ***1*** or Benzophenone

In a flame-dried apparatus and under argon atmosphere, a solution of *cis*-propenylbromide (0.425 mL, 0.005 mol) in anhydrous THF (30 mL) was added dropwise to magnesium turnings (0.005 mol) in THF (5.0 mL). After a gentle warming the Grignard formation was observed and the reaction was allowed until the magnesium was consumed. The resulting solution was withdrawn with a graduate syringe and a half amount was inserted in a dropping funnel and the other half in another one. Each funnel was connected with a flame-dried round-bottomed flask in which 0.00025 mol of ketone were dissolved in 5.0 mL of anhydrous THF and kept at −70 °C. After about 15 min from the addition of the Grignard solution, the reaction was quenched with aqueous (NH_4_)_2_SO_4_ and extracted with diethyl ether. The organic layer was dried over anhydrous Na_2_SO_4,_ and the solvent was removed after filtration. The reaction mixture was analyzed through ^1^H-NMR spectroscopy. The reaction products were recognized by comparison with literature data in case of reaction with benzophenone [25] and with data obtained in experiments reported in Table 1, entries 5 and 6.

### 3.5. X-ray Diffraction Data

#### Crystallographic Data Collection and Structure Determination

The X-ray intensity data of **1**, **5**, **8**, and **11** were measured with a Bruker Apex II CCD diffractometer. The cell dimensions and the orientation matrix were initially determined by a least squares refinement of reflections measured in three sets of 20 exposures, which were collected in three different ω regions, and eventually refined against all data. A full sphere of reciprocal space was scanned in 0.3°ω steps. The SMART [29] software was used to collect frames of data, index reflections, and determine the lattice parameters. The collected frames were then processed for integration by the SAINT program, [29] and an empirical absorption correction was applied with SADABS [30]. The structures were solved by direct methods (SIR2004) [31] and subsequent Fourier syntheses and refined by full-matrix least-squares techniques on F^2^ (SHELXTL) [32] with anisotropic thermal parameters for all non-hydrogen atoms. All hydrogen atoms were added in calculated positions in the final stage of refinement and refined with U(H) = 1.2 U_eq_(C) and allowed to ride on their carrier atoms. In the asymmetric unit of **5** there are two independent molecules. In **11.2H_2_O** two water molecules are present in the asymmetric unit. Crystal data and details of the data collections for **1**, **5**, **8**, and **11** are reported in Table 3.

CCDC 1587827 (for **1**), 1587828 (for **5**), 1587829 (for **8**), and 1587830 (for **11**) contain the Supplementary crystallographic data for this paper. These data can be obtained free of charge from The Cambridge Crystallographic Data Centre (https://www.ccdc.cam.ac.uk/, Tel.: +44 (0)1223 336408, Fax: +44 (0)1223 336033).

## 4. Conclusions

The regioselectivity of the attack of vinyl Grignard reagents to bis(benzothiazolyl)ketone (**1**) strongly depends on the alkene geometry and on its substitution grade: the exclusive attack to the carbonylic oxygen atom occurring with vinyl Grignard reagents **2a** and **2b** indicates that the absence of substituents on the vinyl moiety or the presence of an alkyl substituent on the same carbon atom bound to the magnesium might play a key role in driving the regioselectivity of the attack towards the *O*-alkylation. In the presence of a methyl group bound to the carbon atom in β-position with respect to the Mg atom an almost complete *O*-alkylation was observed; the alkene was *E* stereoisomer. However, the *Z*-isomer undergoes both *C*-and *O*-alkylation. From the current data it emerges that the regioselectivity of the addition of vinyl Grignard reagents to **1** cannot be explained only by the steric effect or the geometry of the vinyl moiety but that it can be due to the concomitance of different effects on the mechanistic pathway. The latter was also investigated by reaction between **1** and vinylmagnesium bromide (and 2-methyl-1-propenylmagnesium bromide) under different experimental conditions and in the presence of radical scavengers: the results observed suggest a mechanistic pathway in the *O*-alkylation involving an intermediate bearing radical oxygen atom and negative charge in benzylic position. By comparing the results from the reaction between 2-propenylmagnesium bromide and benzophenone or bis(2-benzothiazolyl)ketone with the literature data we infer that a polar mechanism is likely involved in the formation of carbinols.

The X-ray diffraction studies on four bis(heteroaryl)ketones, i.e., bis(benzothiazolyl)ketone (**1**), bis(2-thiazolyl)ketone (**5**), bis(2-benzoxazolyl)ketone (**8**), and bis(1-methylbenzimidazolyl)ketone (**11**) and comparison of the structural data supported the speculation that the ability in delocalizing the negative charge in the benzylic position of intermediate may play a key role in driving the attack of the vinyl Grignard reactant towards the *O*-alkylation.

## Figures and Tables

**Table 1:** Reaction of **1** with vinyl Grignard reagents **2a**–**h**. ^a^.

Entry	Grignard Reagent	*C*-Alkylation Product (%)	*O*-Alkylation Product (%)
1	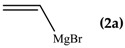	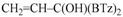 **3a** ^b,c^ n.d. ^d^	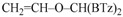 **4a** ^b^ > 97 ^c^
2	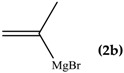	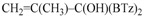 **3b** ^b,c^ n.d. ^d^	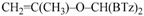 **4b** ^b^ > 97 ^c^
3	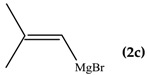	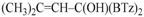 **3c** 57 ^b,c^	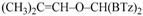 **4c** ^b^ 43 ^c^
4	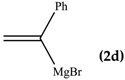	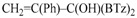 **3d** 70 ^c^	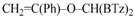 **4d** 30 ^c^
5	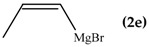	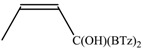 **3e** 70 ^c,e^	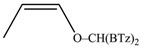 **4e** 30 ^c,e^
6	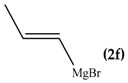	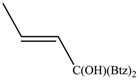 **3f** 5	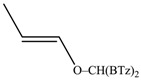 **4f** 95 ^c^
7	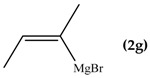	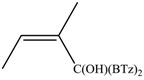 **3g** 90 ^c^	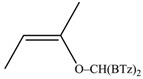 **4g** 10 ^c^
8	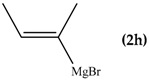	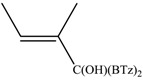 **3h** 5 ^f^	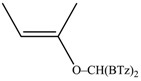 **4h** n.d. ^d^

^a^ Reactions were carried out in tetrahydrofurane (THF) at −70 °C with an equimolar reactants ratio within 15 min; ^b^ Data from reference 10; ^c^ Relative ratio calculated from ^1^H-NMR of crude reaction mixture; ^d^ n.d. means not detected; ^e^ In this case (entry 5), due to the reaction with **2f**, produced by isomerization during the Grignard preparation, crude reaction mixture contains carbinol **3f** and vinyl ether **4f** (in about 40% relative molar ratio with respect to the mixture of **3e** and **4e**); ^f^ In this case, the crude reaction mixture contains mostly the alcohol **3g**, coming from **2g**, in turn formed by isomerization during Grignard preparation.

**Table 2:** Reactions of 1 with 2a and 2c under different experimental conditions. ^a^

Entry	Vinyl Reagent	Solvent	Reaction Time (min)	Radical Scavenger	Conversion% ^b^	*C*-Alkylation Product (%) ^c^	*O*-Alkylation Product (%) ^c^
1	**2a**	THF	10	-	80	**3a** (n.d.) ^d^	**4a** (pres.) ^e^
2	**2a**	THF	10	TEMPO (1.0 eq.)	60	**3a** (n.d.) ^d^	**4a** (pres.) ^e^
3	**2c**	THF	10	-	80	**3c** (67)	**4c** (33)
4	**2c**	THF	10	TEMPO (0.5 eq.)	50	**3c** (67)	**4c** (33)
5	**2c**	THF	10	TEMPO (1.0 eq.)	30	**3c** (pres.) ^e^	**4c** (n.d.) ^d^
6	**2c**	THF	15–30–45–90	-	80	**3c** (67) ^c,f^	**4c** (33) ^c,f^
7	**2a**	Et_2_O	60	-	28	**3a** (50)	**4a** (50)

^a^ Reactions carried out at −70 °C with equimolar amount of reagents; ^b^ calculated with respect to **1**; ^c^ relative ratio between *C*- and *O*-alkylation product calculated from ^1^H-NMR of the crude reaction mixture; ^d^ n.d. means not detected; ^e^ Pres. means presence of the product; we selected to indicate ‘pres’. because the absence of carbinol does not permit to give **3**:**4** ratio value; ^f^ This relative ratio remained unchanged.

**Table 3:** Crystal data and structure-refinement details for **1**, **5**, **8**, and **11.2H_2_O**.

Compound	1	5	8	11.2H_2_O
Empirical formula	C_15_H_8_N_2_OS_2_	C_7_H_4_N_2_OS_2_	C_15_H_8_N_2_O_3_	C_17_H_14_N_4_O.2H_2_O
Formula. weight	296.35	196.24	264.23	326.35
Temperature	273(2) K	273(2) K	273(2) K	273(2) K
Wavelength	0.71073 A	0.71073 A	0.71073 A	0.71073 A
Crystal system	orthorhombic	Monoclinic	Monoclinic	Monoclinic
Space group	*Pna2_1_*	*C_c_*	*P2_1_*/*c*	*P2_1_*/*n*
*a*, Å	18.3273(12)	3.7993(3)	13.1814(14)	7.3636(7)
*b*, Å	16.2782(11)	29.361(2)	11.9694(13)	15.5117(14)
*c*, Å	4.2718(3)	13.7502(10)	7.6380(8)	14.4556(13)
α, °	90	90	90	90
β, °	90	91.769(1)	100.217(2)	103.544(1)
γ , °	90	90	90	90
Volume, Å^3^	1274.43(15)	1533.1(2)	1186.0(2)	1605.2(3)
*Z*, *Dc*, Mg m^−3^	4, 1.545	8, 1.700	4, 1.480	4, 1.350
μ(Mo-K_α_), mm^−1^	0.412	0.636	0.106	0.095
F(000)	608	800	544	688
Crystal size, mm	0.28 × 0.15 × 0.15	0.20 ×0.15 × 0.12	0.35 × 0.30 × 0.20	0.20 × 0.15 × 0.10
θ limits, °	1.67–28.62	1.39–28.66	1.57–27.989	1.96–26.24
Reflections collected	10765	6588	9953	12377
Unique obs. Reflections [F_o_ > 4σ(F_o_)]	3045 [R(int) = 0.0188]	3461 [R(int) = 0.0183]	2785 [R(int) = 0.0190]	3230 [R(int) = 0.0242]
Goodness-of-fit on F^2^	1.076	1.037	0.993	1.090
R_1_ (F)^a^, wR_2_ (F^2^) [I > 2σ(I)]	0.0255, 0.0681	0.0239, 0.0592	0.0348, 0.0883	0.0448, 0.1243
R_1_ (F)^a^, wR_2_ (F^2^) (all data)	0.0279, 0.0691	0.0254, 0.0597	0.0473, 0.0947	0.0682, 0.1366
Largest diff. peak and hole, e. Å^−3^	0.291 and −0.155	0.339 and −0.337	0.165 and −0.134	0.355 and −0.195

^a^ R_1_ = Σ||F_o_| − |F_c_||/Σ|F_o_|; ^b^ wR_2_ = [Σw(F_o_^2^ − F_c_^2^)^2^/Σw(F_o_^2^)^2^]^1/2^ where w = 1/[σ^2^(F_o_^2^) + (*a*P)^2^+ *b*P] where P = (F_o_^2^ + F_c_^2^)/3.

## Supplementary Materials

Supplementary materials are available online.
