# Supplementary material for: Regioselectivity in Reactions between Bis(2-benzothiazolyl)ketone and Vinyl Grignard Reagents: C- versus O-alkylation—Part III
Source: Molecules. 2018 Jan 15;23(1):171. doi: 10.3390/molecules23010171 (PMC6017936; doi:10.3390/molecules23010171)

SUPPORTING INFORMATION

Regioselectivity in reactions between Bis(2-benzothiazolyl)ketone and vinyl Grignard reagents: *C*- versus *O*- alkylation. Part III[&]

Carla Boga [1,*] Silvia Bordoni[1], Lucia Casarin[1], Gabriele Micheletti[1,*], and Magda Monari[2]

1 Department of Industrial Chemistry 'Toso Montanari', Alma Mater Studiorum – Università di Bologna Viale Del Risorgimento 4 - Bologna 40136, Italy; luciacasarin2000@yahoo.it; gabriele.micheletti3@unibo.it; silvia.bordoni@unibo.it; carla.boga@unibo.it.

3 Department of Chemistry 'Giacomo Ciamician', Alma Mater Studiorum – Università di Bologna Via Selmi 2 - Bologna 40126, Italy; magda.monari@unibo.it.

* Correspondence: gabriele.micheletti3@unibo.it (G.M.); carla.boga@unibo.it (C.B.). Tel.: +39-051-209-3616 (C.B.)

Content **Page**

^{1}H NMR, ^{13}C NMR, mass spectra of new compounds 2

CIF crystallographic data of compounds 1, 5, 8, and 11 14

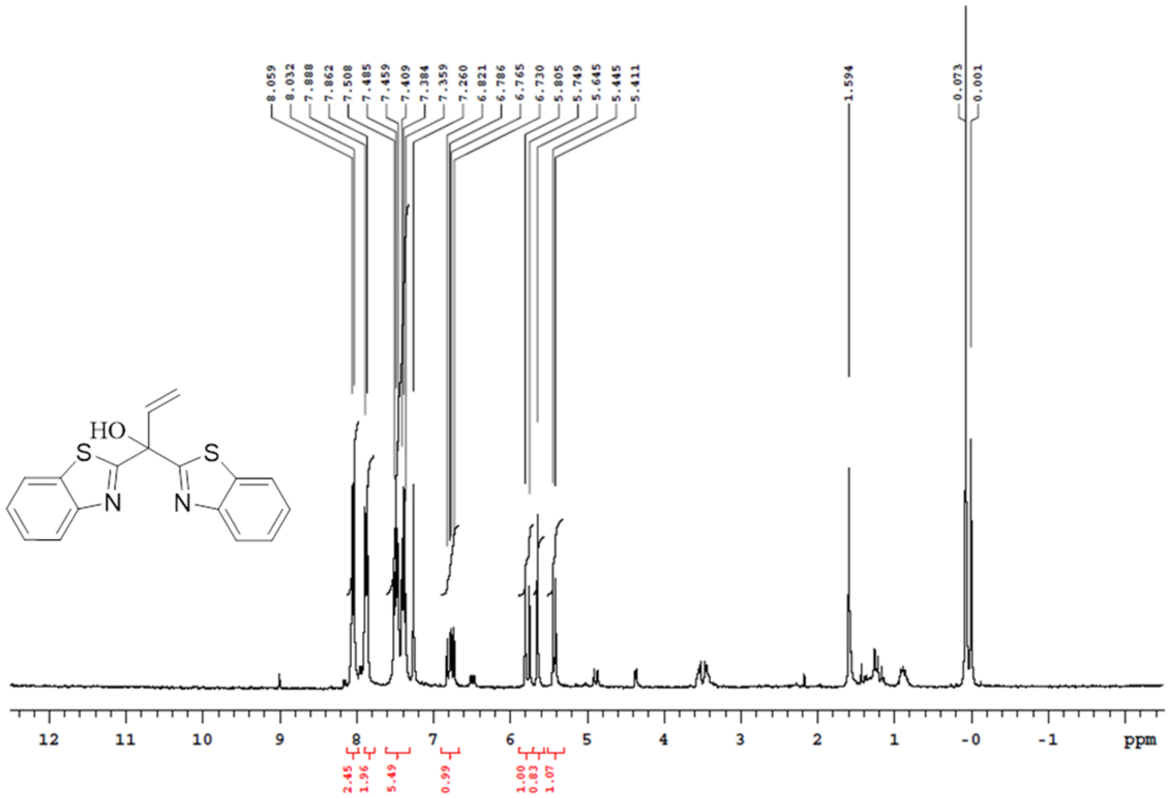

Figure 1. ^{1}H NMR spectrum ($CDCl_3$) of **3a**.

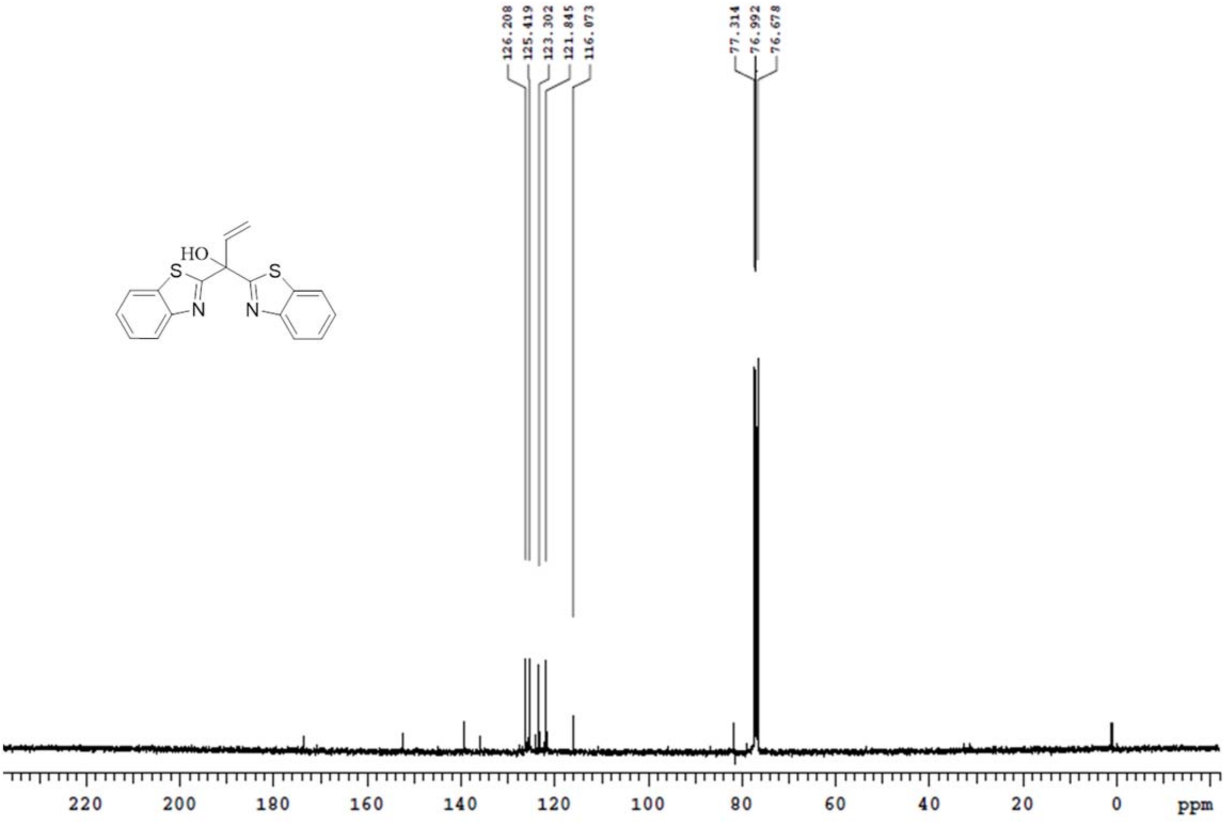

Figure 2. ^{13}C NMR spectrum ($CDCl_3$) of **3a.**

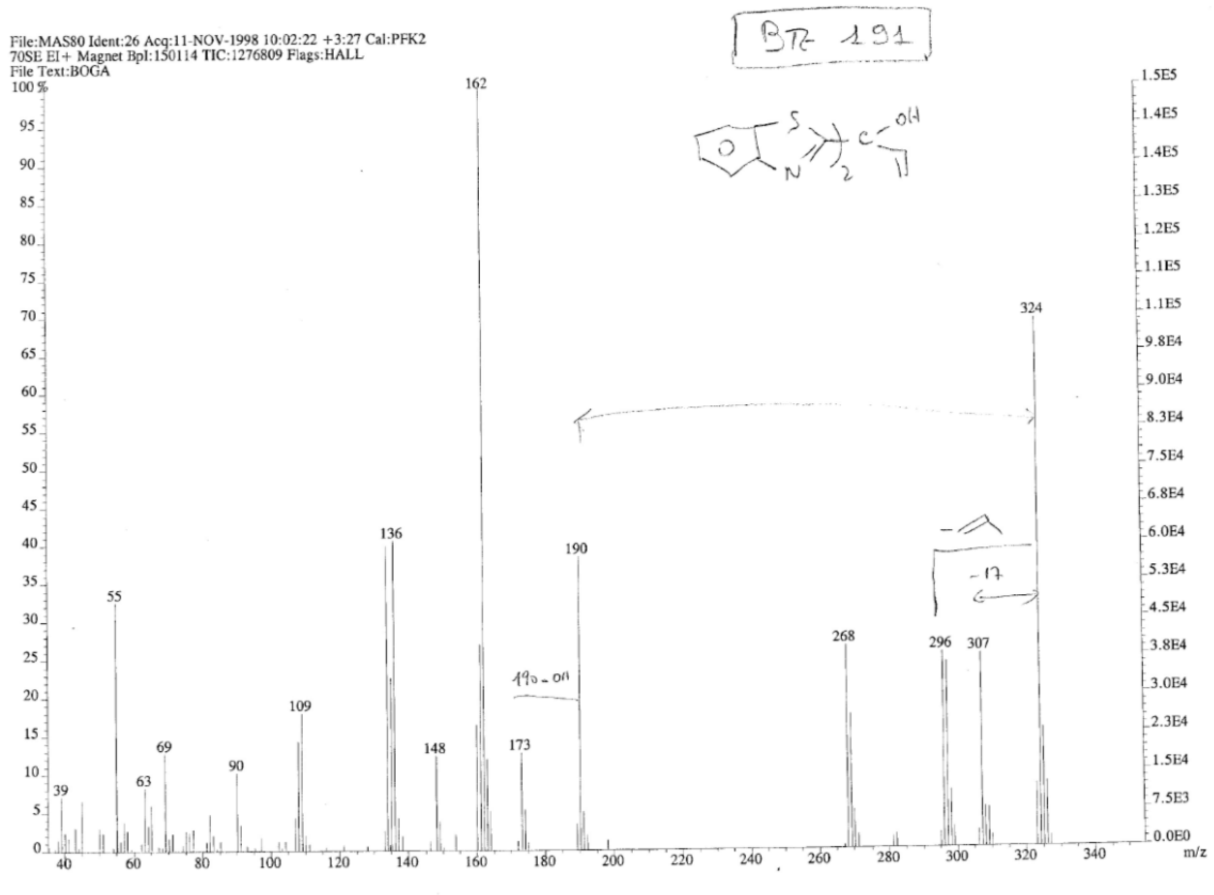

Figure 3. MASS Spectrum of **3a**

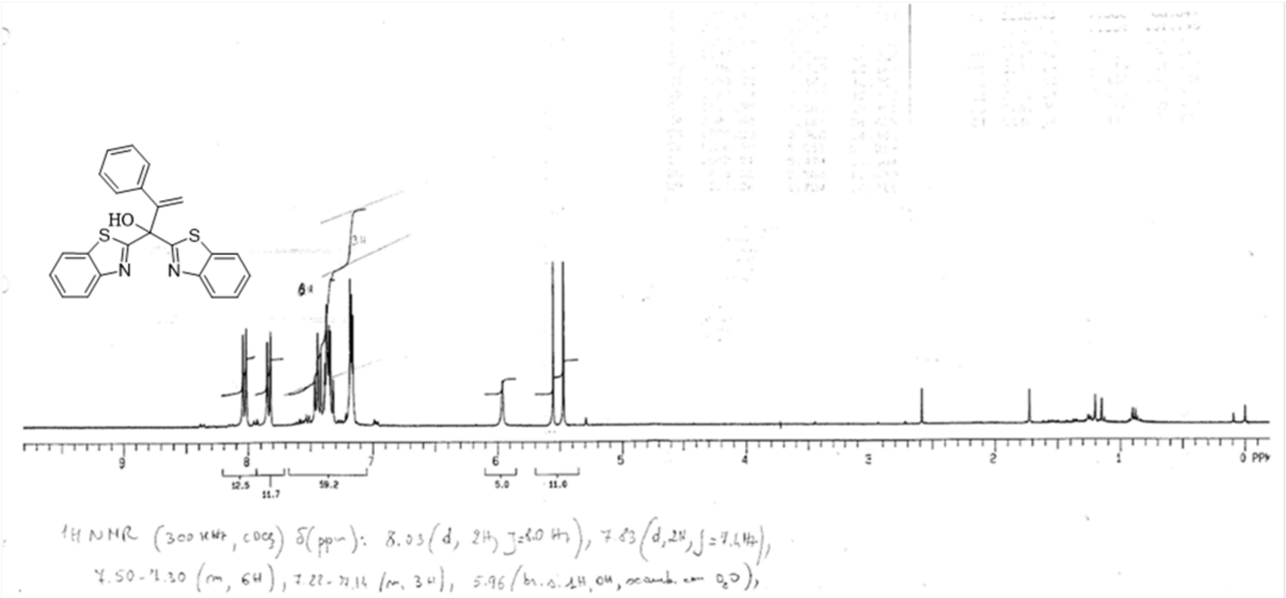

Figure 4. ^{1}H NMR spectrum ($CDCl_3$) of 3d.

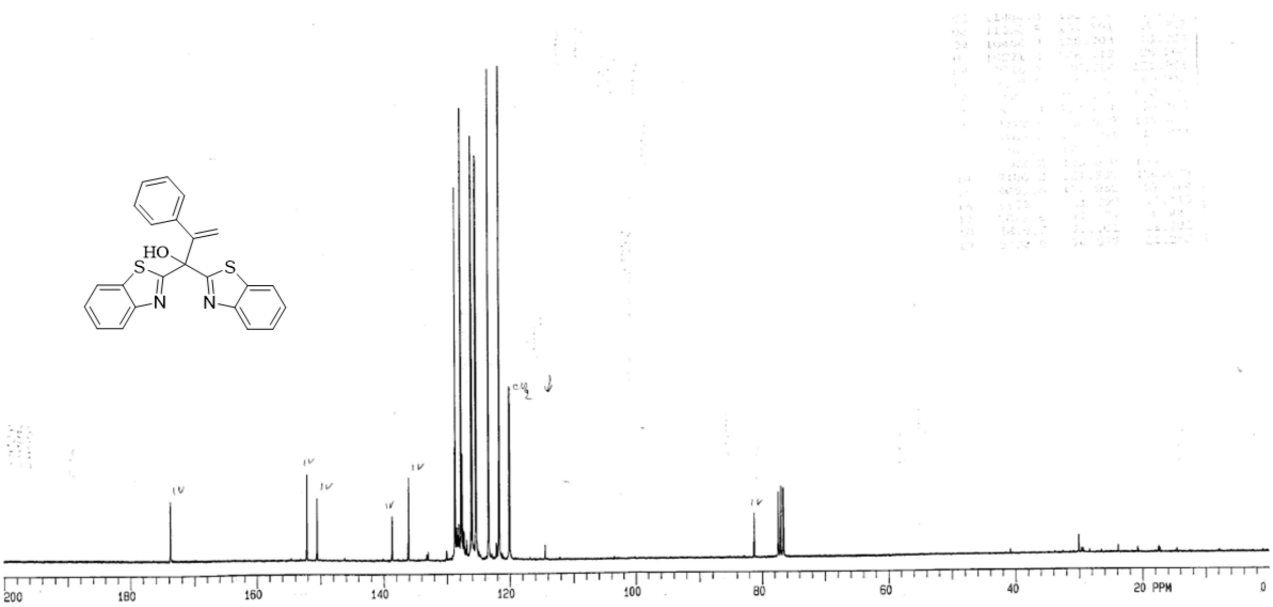

Figure 5. ^{13}C NMR spectrum ($CDCl_3$) of **3d.**

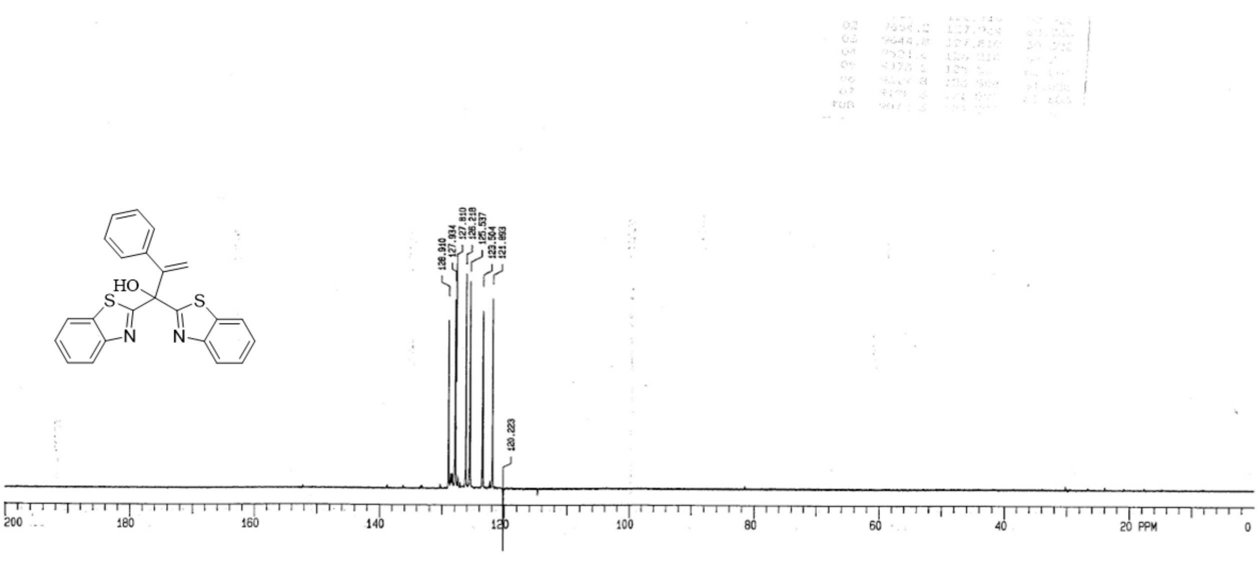

Figure 6. DEPT 135 NMR spectrum ($CDCl_3$) of **3d.**

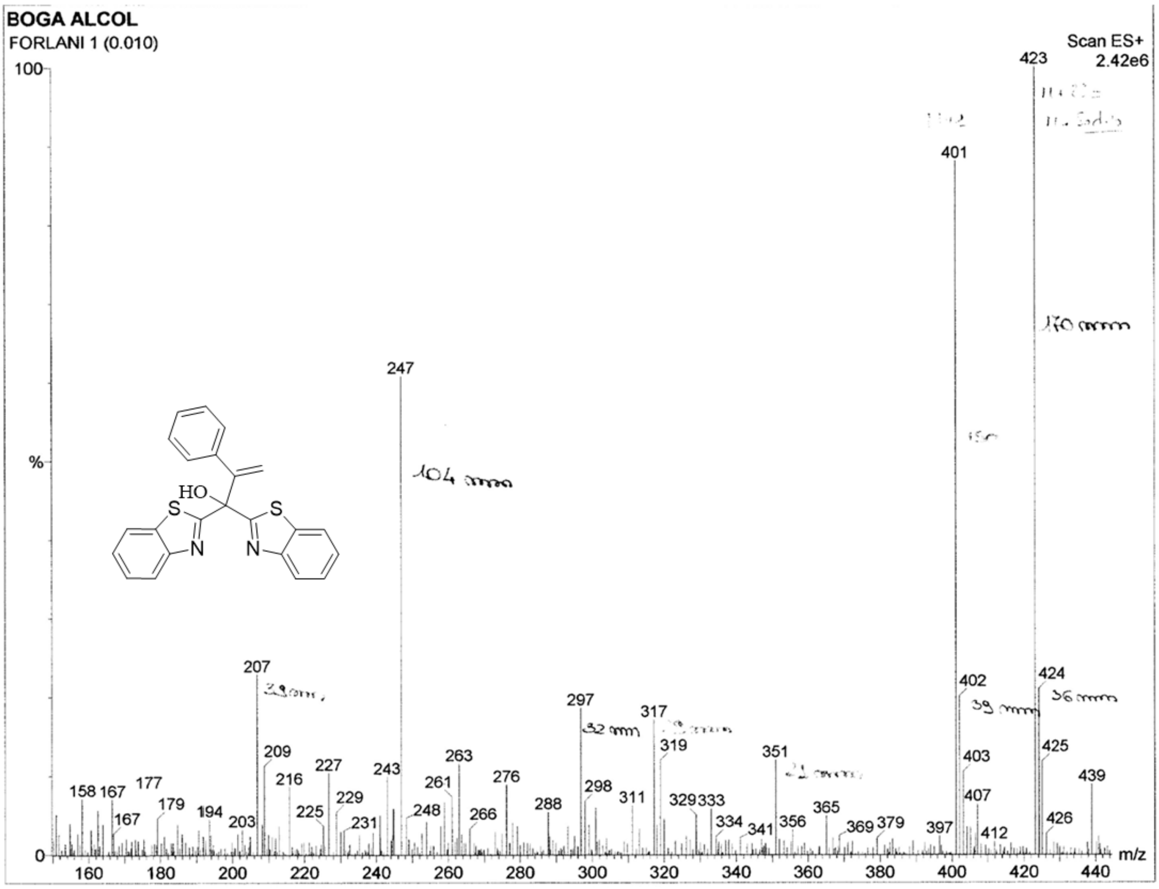

Figure 7. Mass spectrum (ESI[+]) of **3d.**

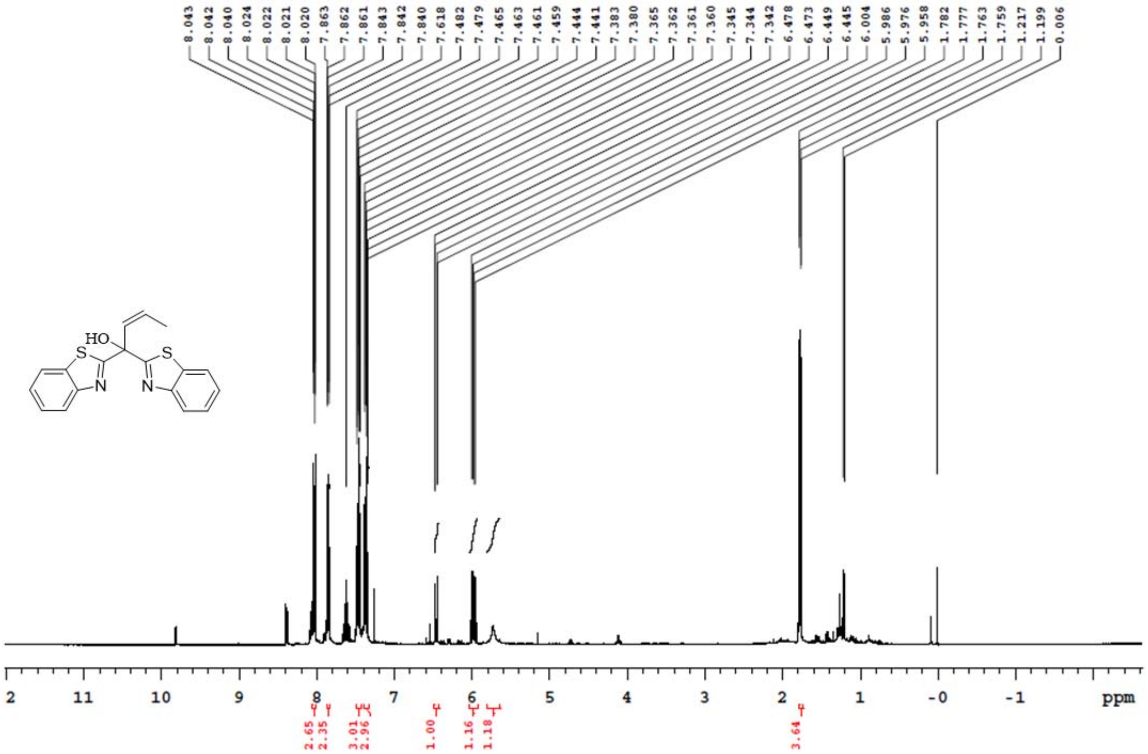

Figure 8. ^{1}H NMR spectrum ($CDCl_3$) of **3e** with traces of **1**.

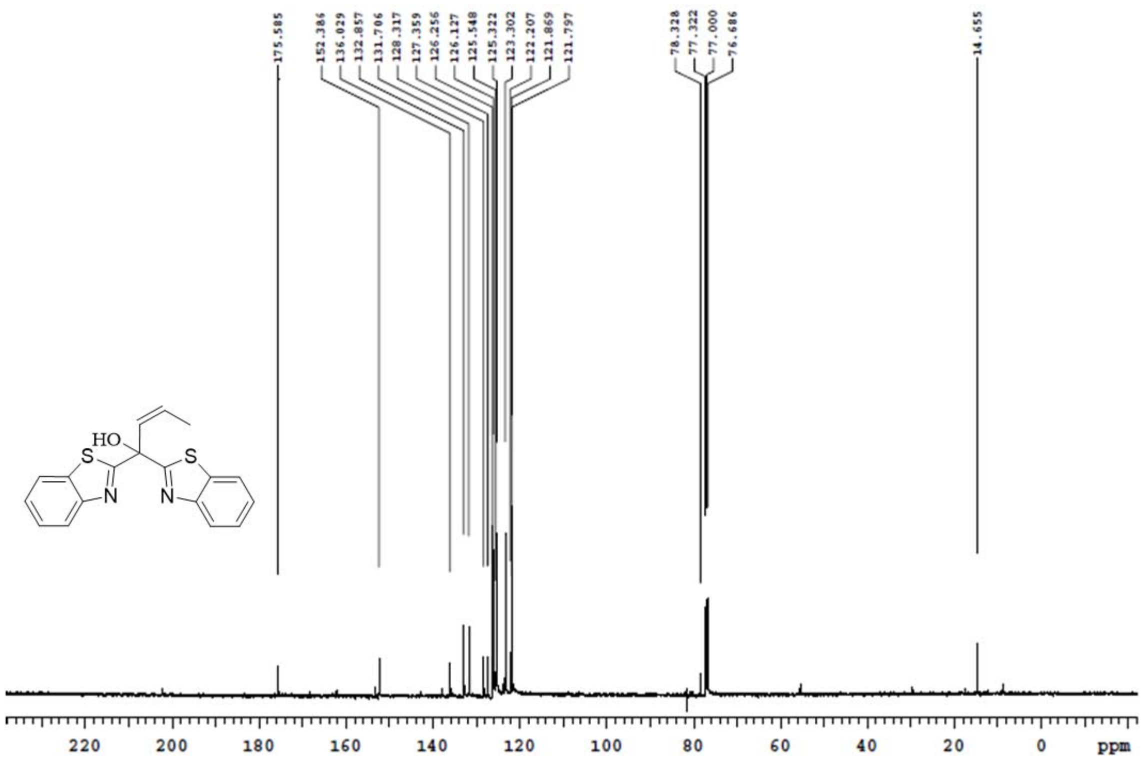

Figure 9. ^{13}C NMR spectrum ($CDCl_3$) of **3e** with traces of **1.**

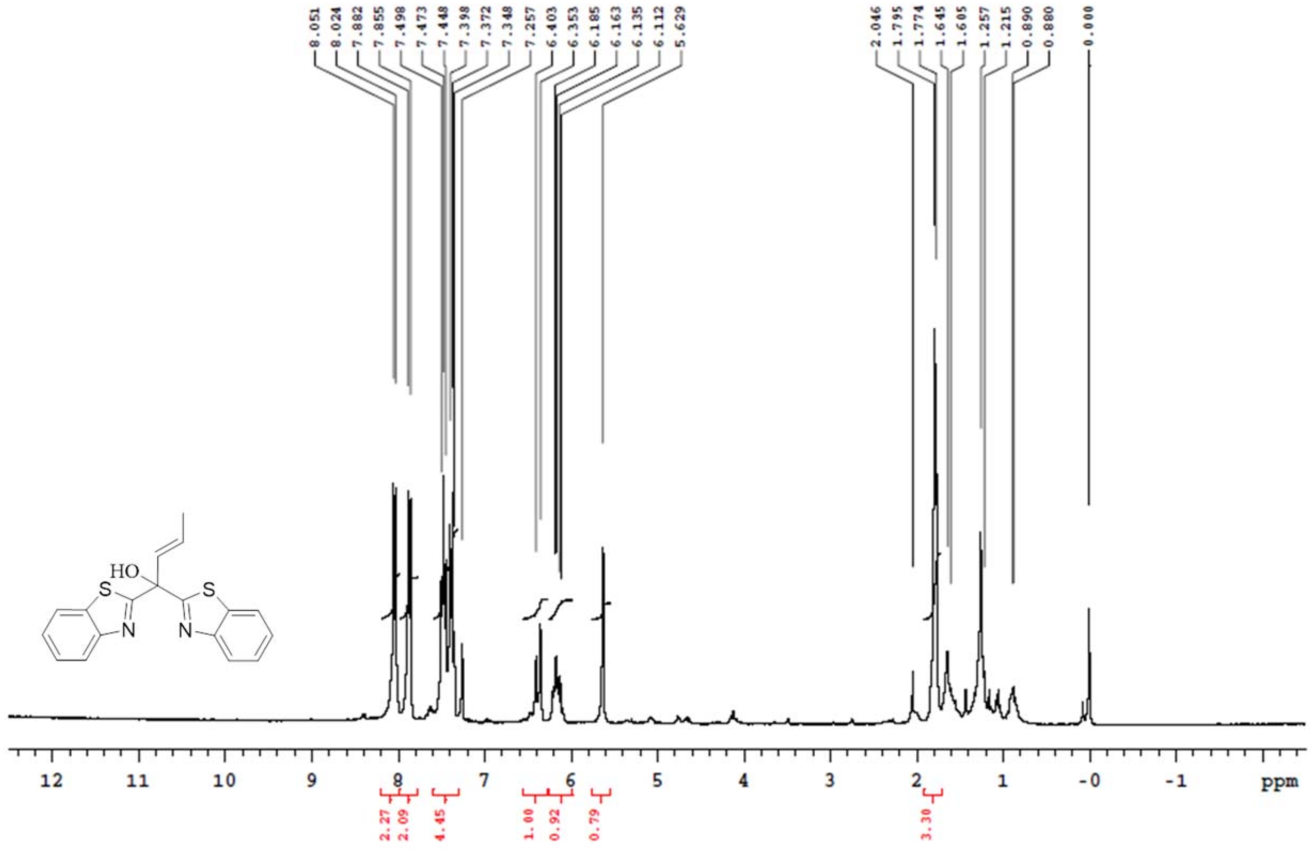

Figure 10. ^{1}H NMR spectrum ($CDCl_3$) **3f** (traces of water and solvents from FC).

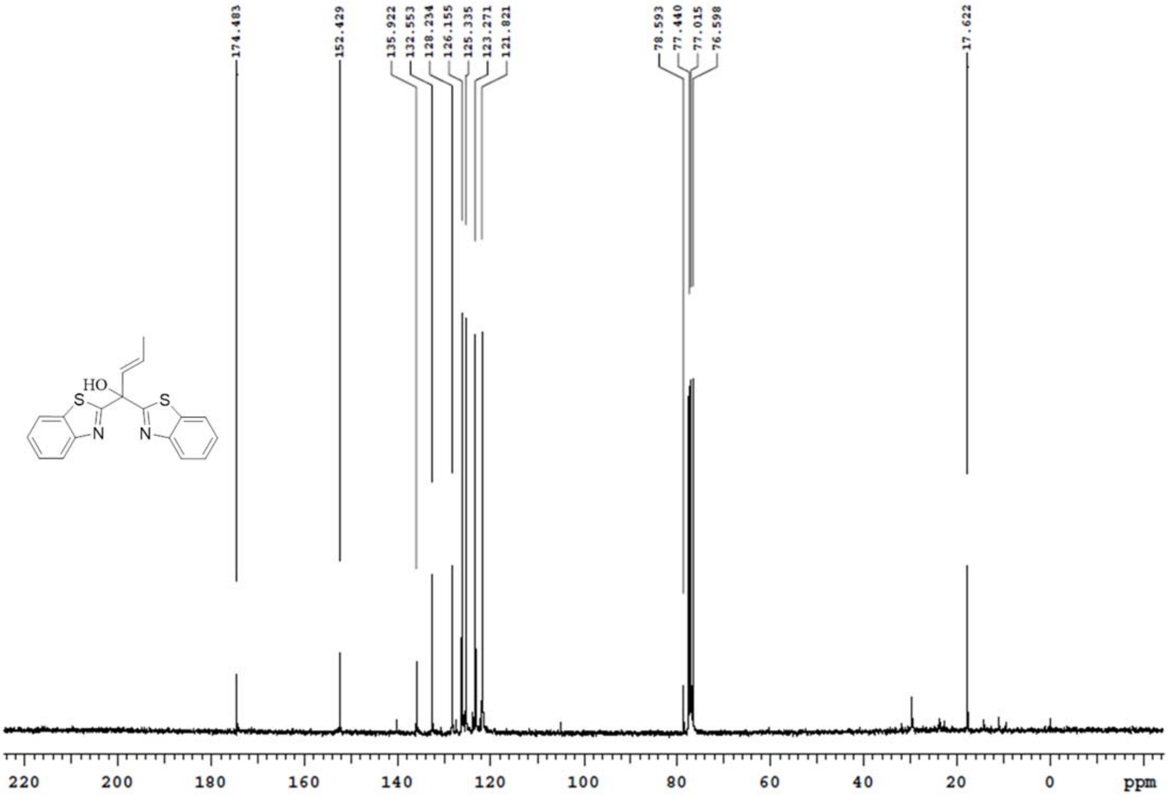

Figure 11. ^{13}C NMR spectrum ($CDCl_3$) of **3f.**

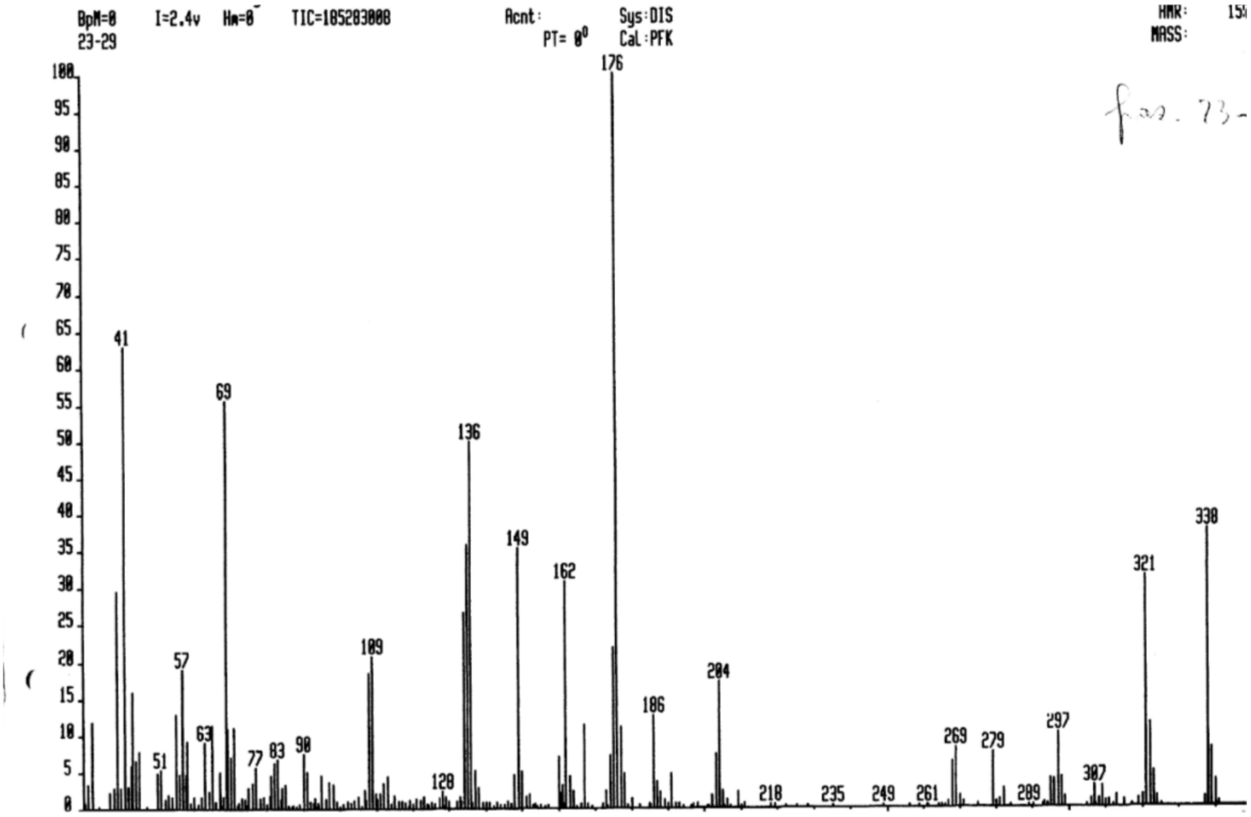

Figure 12. MASS Spectrum of **3f**.

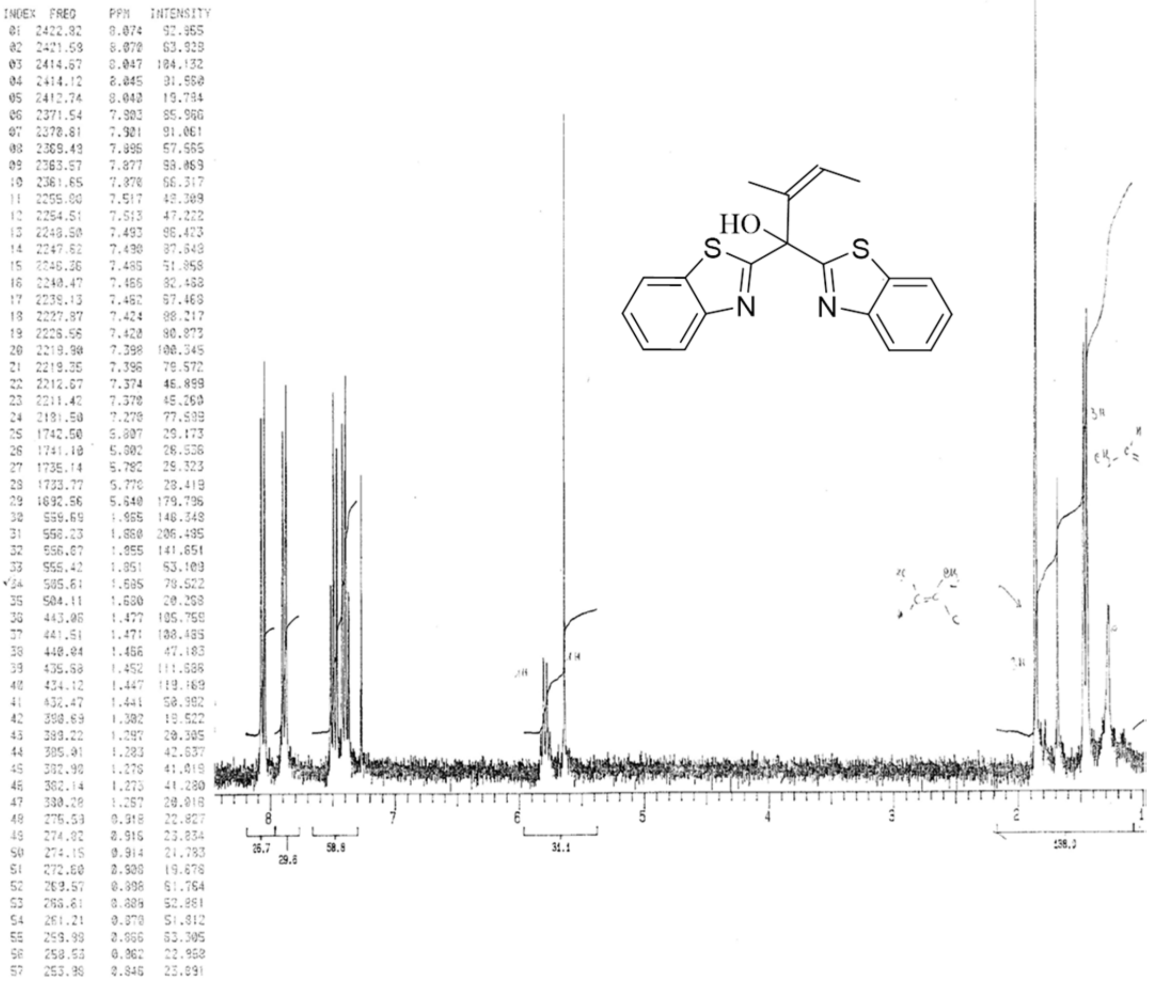

Figure 13. ^{1}H NMR spectrum ($CDCl_3$) of **3g**.

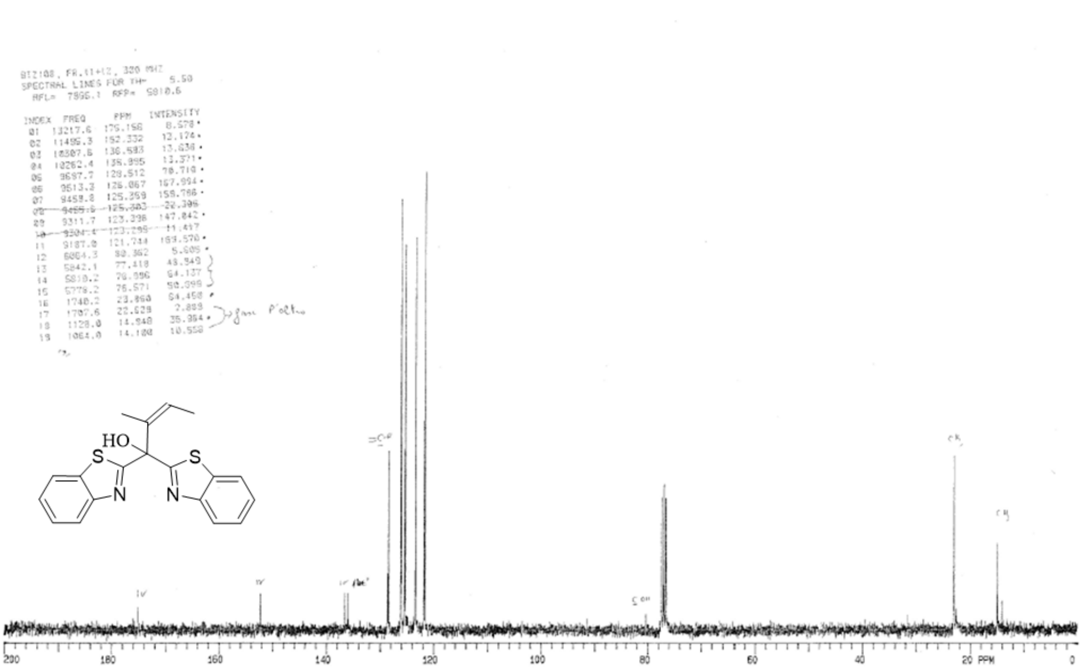

Figure 14. ^{13}C NMR spectrum ($CDCl_3$) of **3g**.

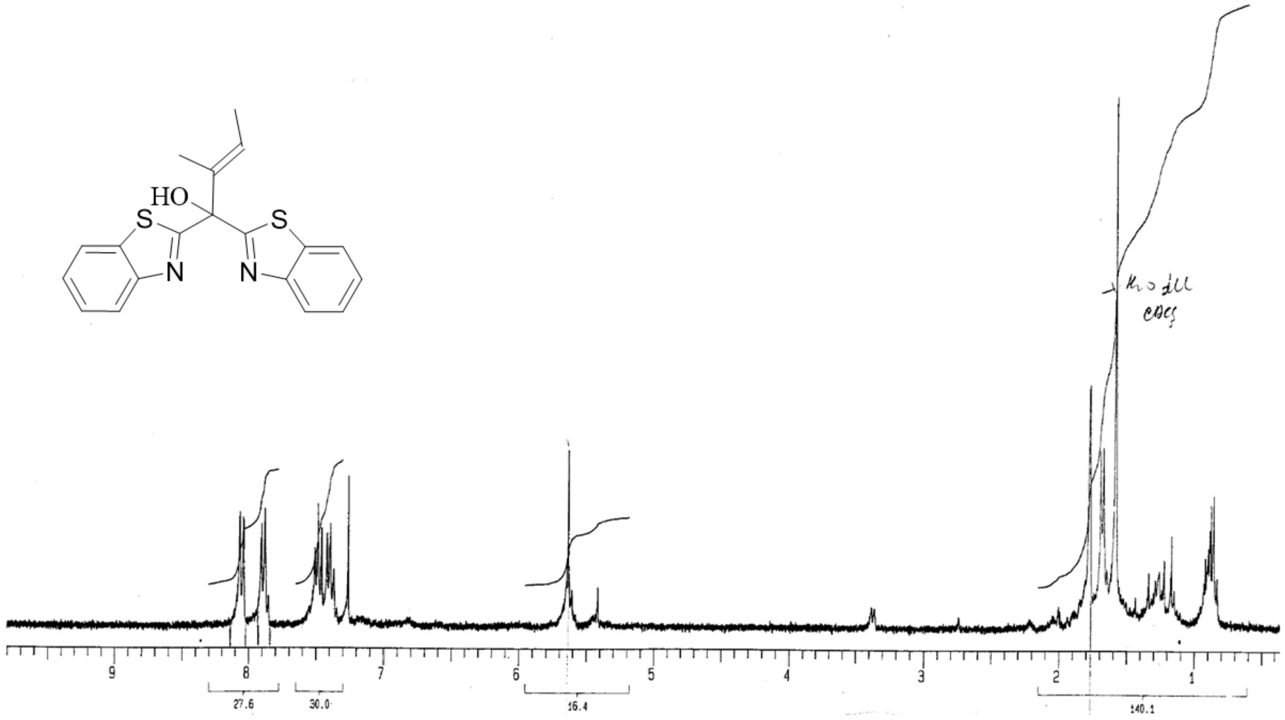

Figure 15. ^{1}H NMR spectrum ($CDCl_3$) of **3h**.

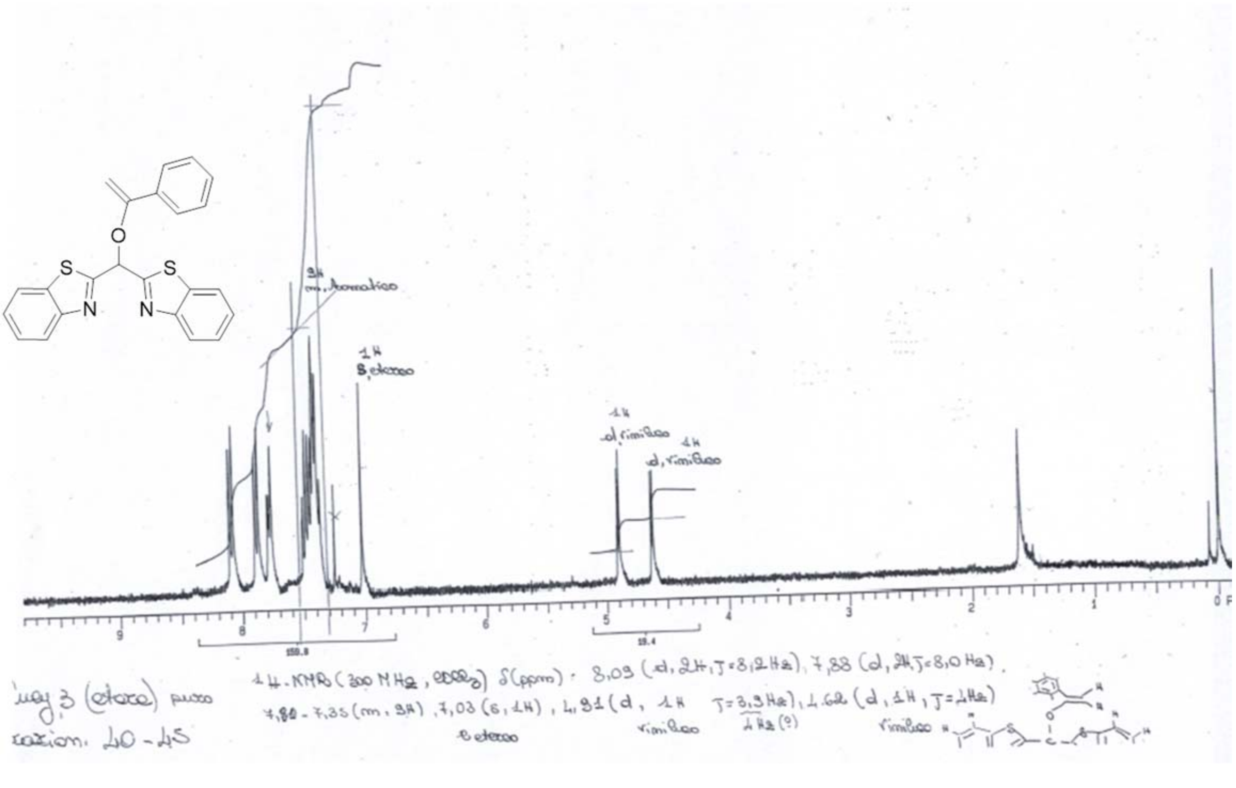

Figure 16. ^{1}H NMR spectrum ($CDCl_3$) of **4d**.

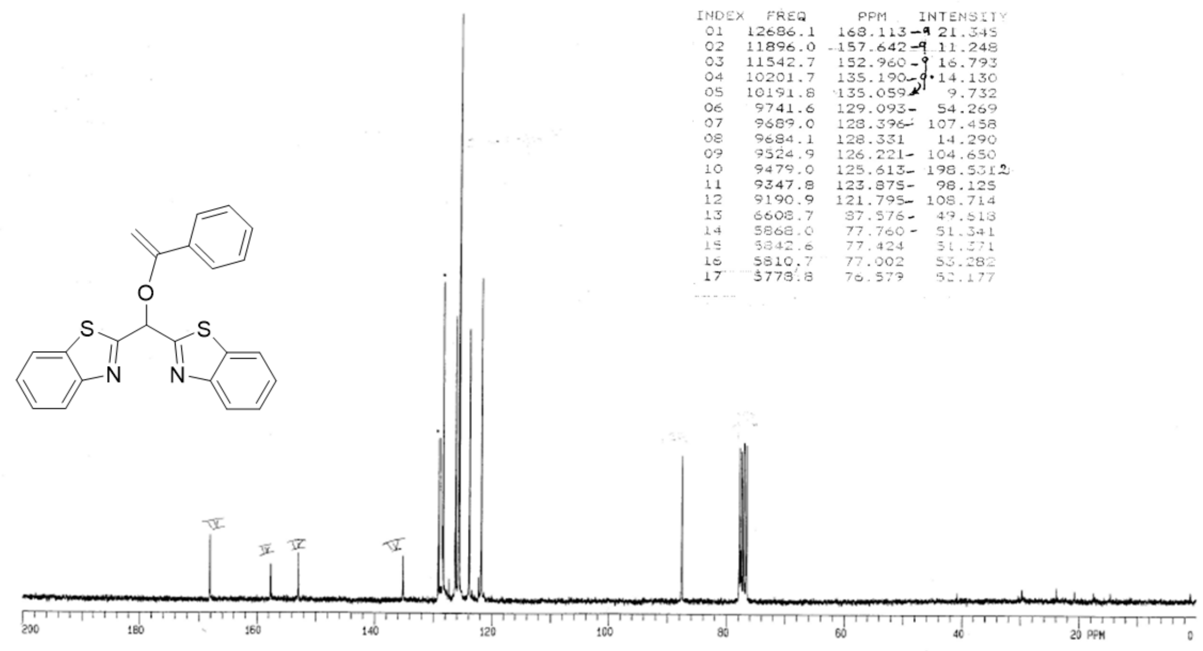

Figure 17. ^{13}C NMR spectrum ($CDCl_3$) of **4d**.

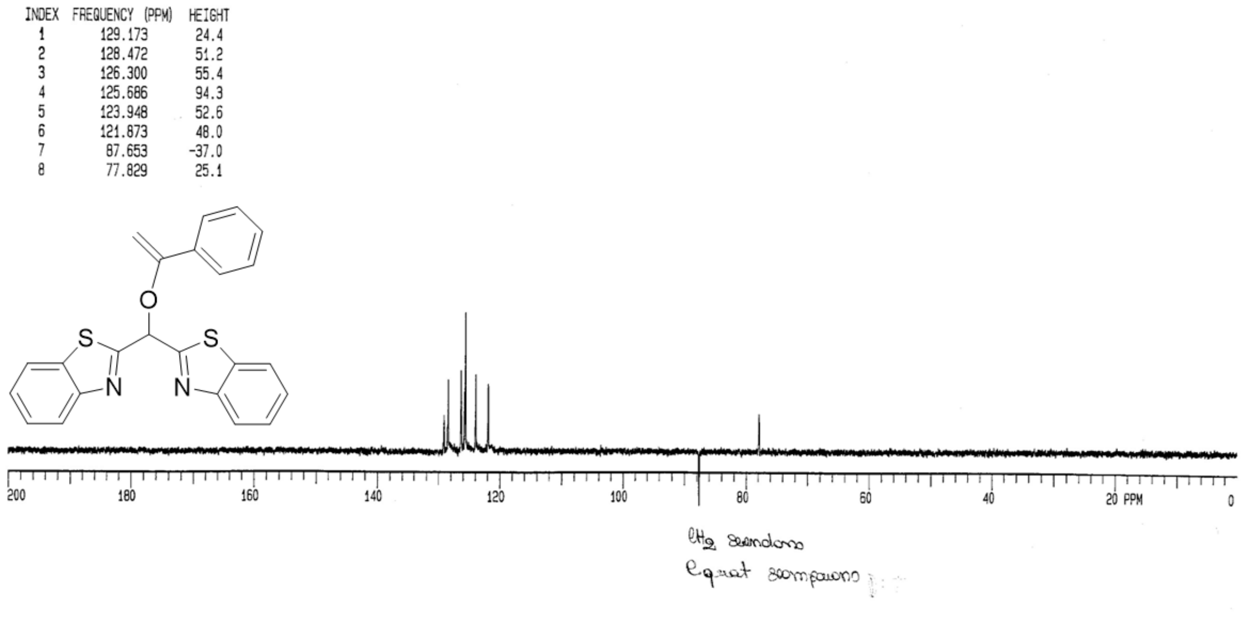

Figure 18. DEPT 135 spectrum ($CDCl_3$) of **4d**.

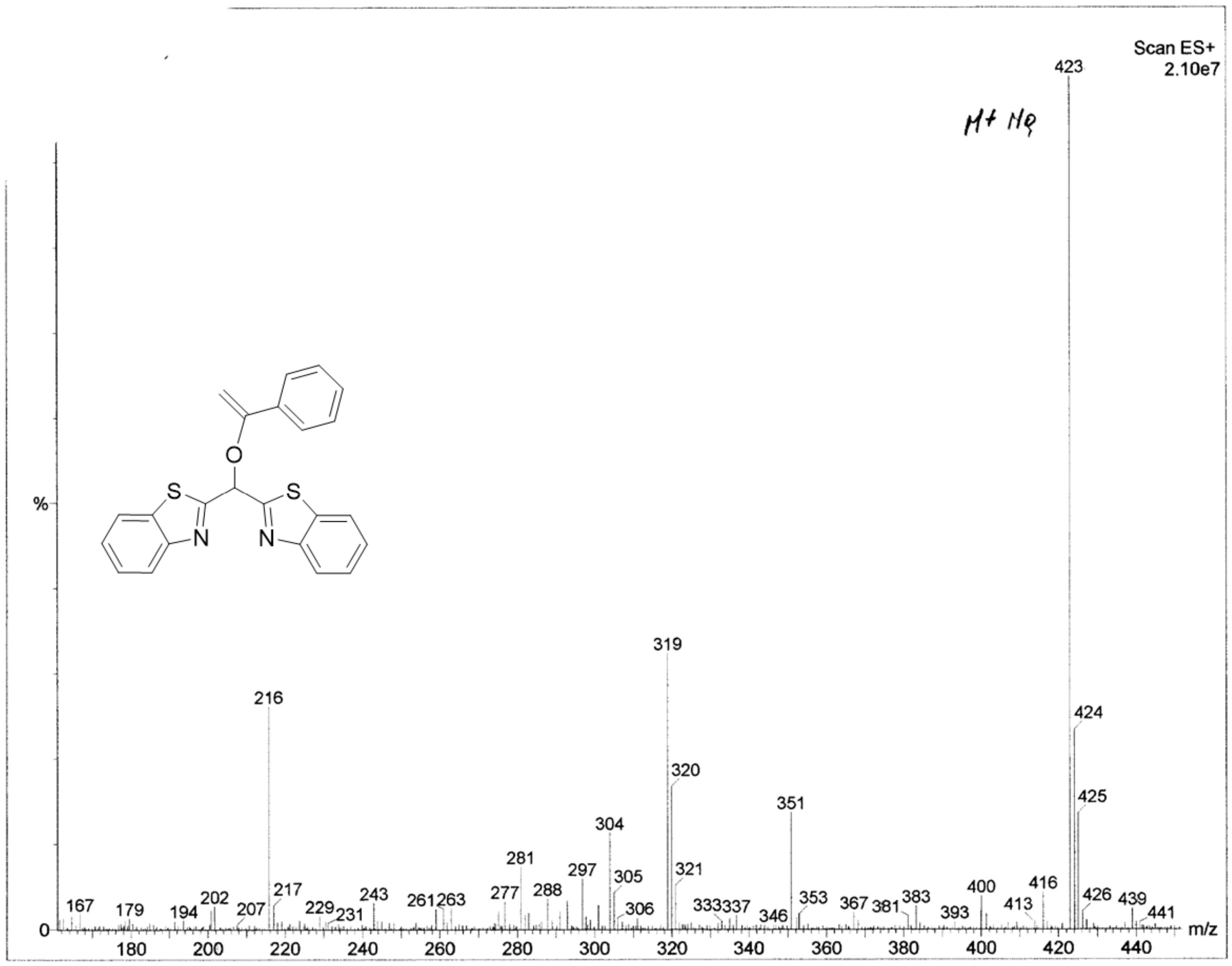

Figure 19. ESI$^+$ Mass spectrum of **4d**.

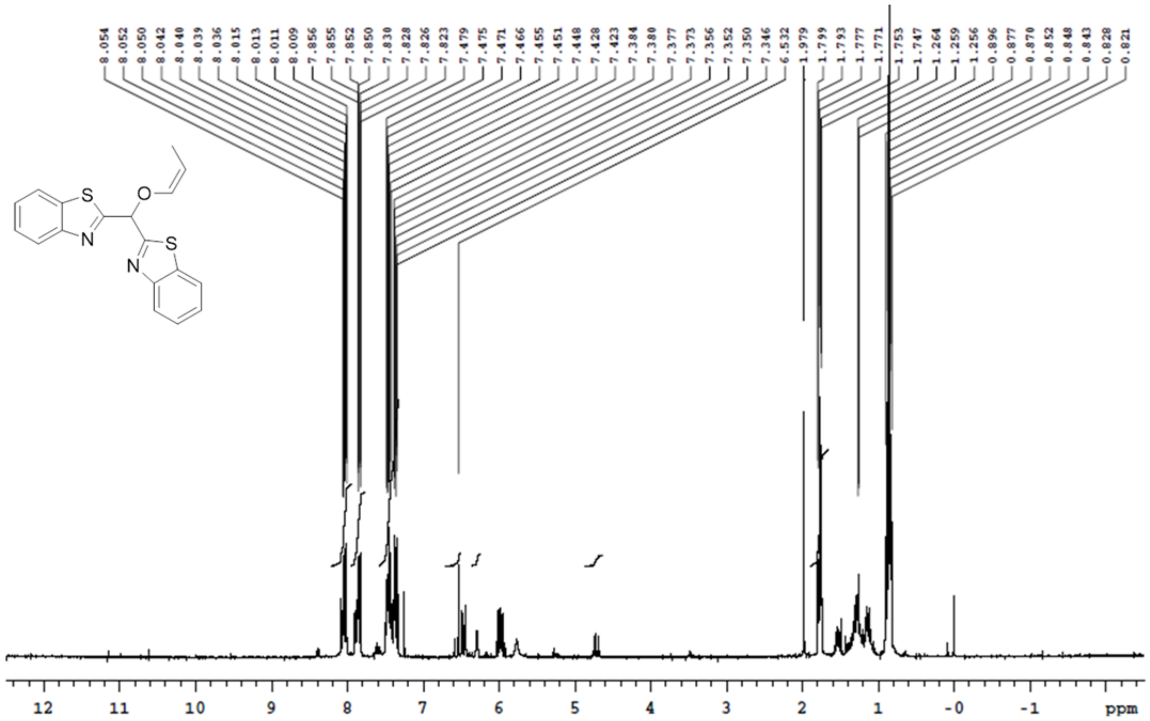

Figure 20. ^{1}H NMR spectrum ($CDCl_3$) of **3e** + **4e**.

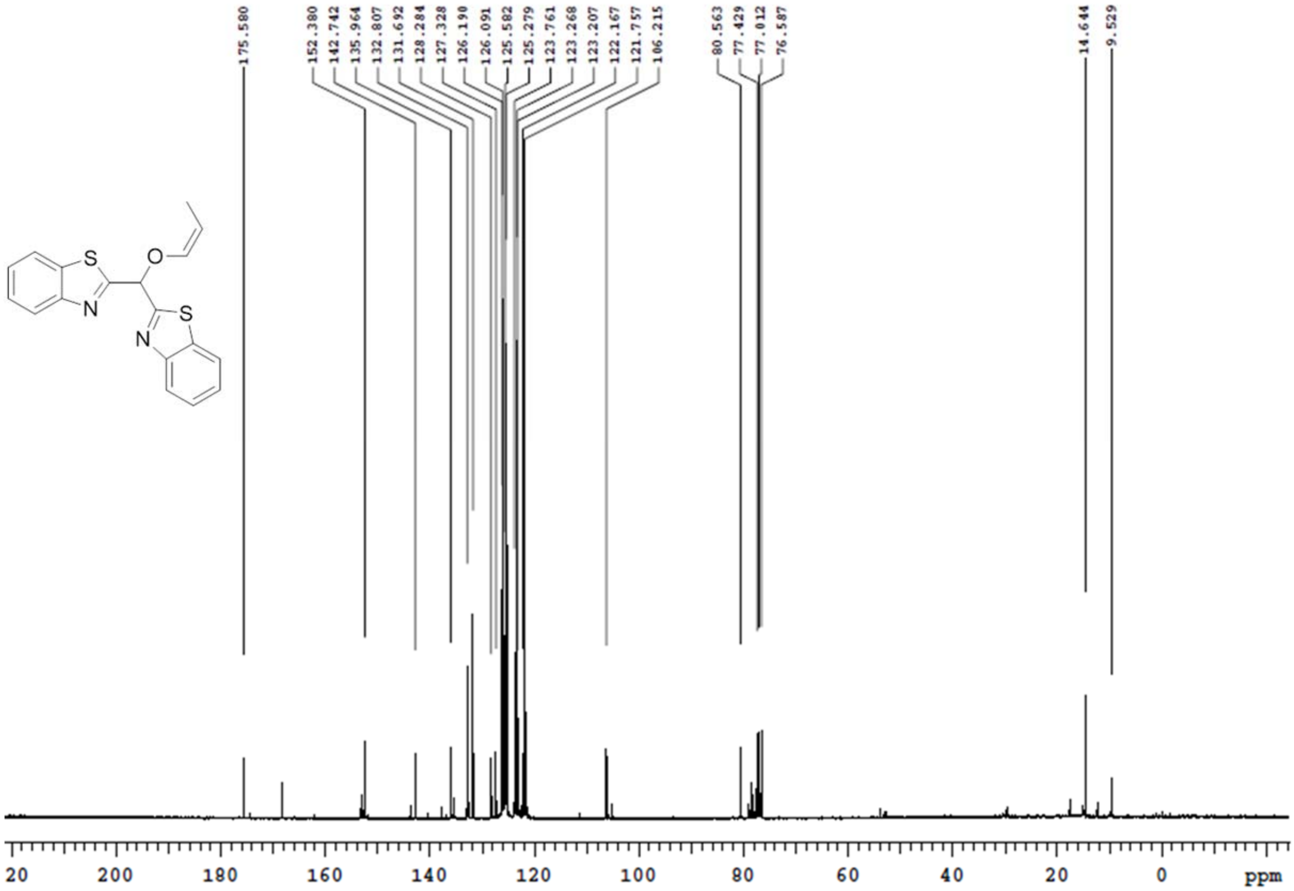

Figure 21. ^{13}C NMR spectrum ($CDCl_3$) of **3e** + **4e**.

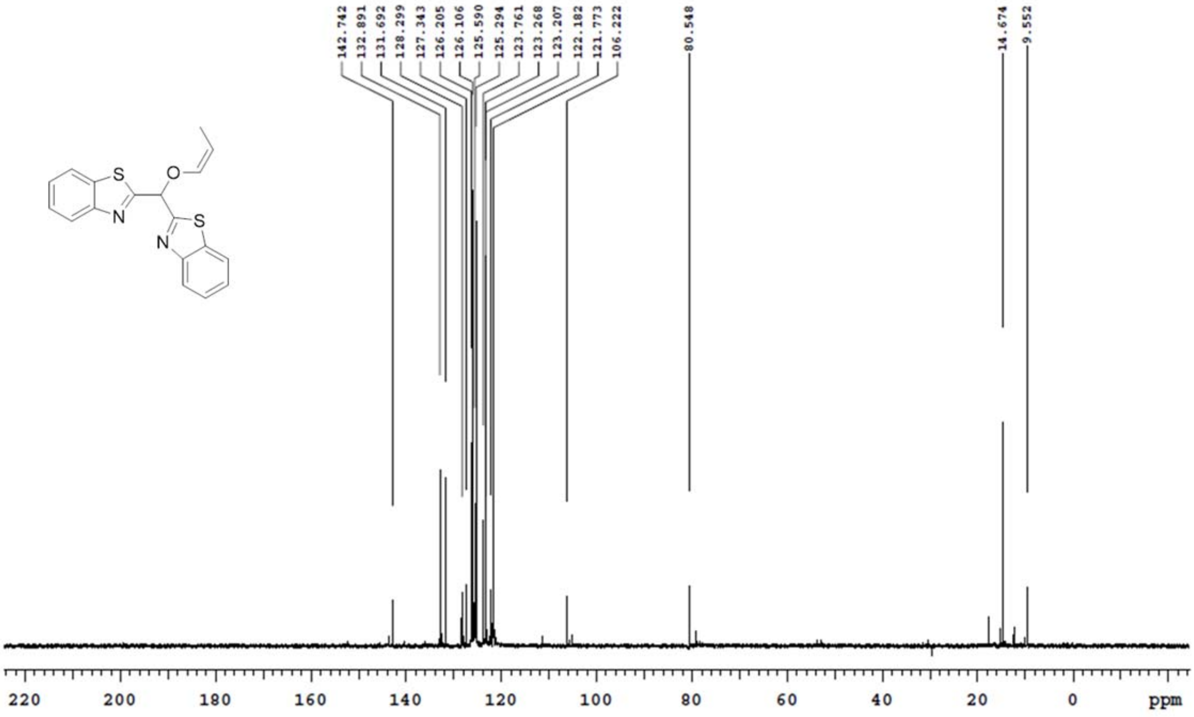

Figure 22. DEPT 135 ($CDCl_3$) of **3e** + **4e**.

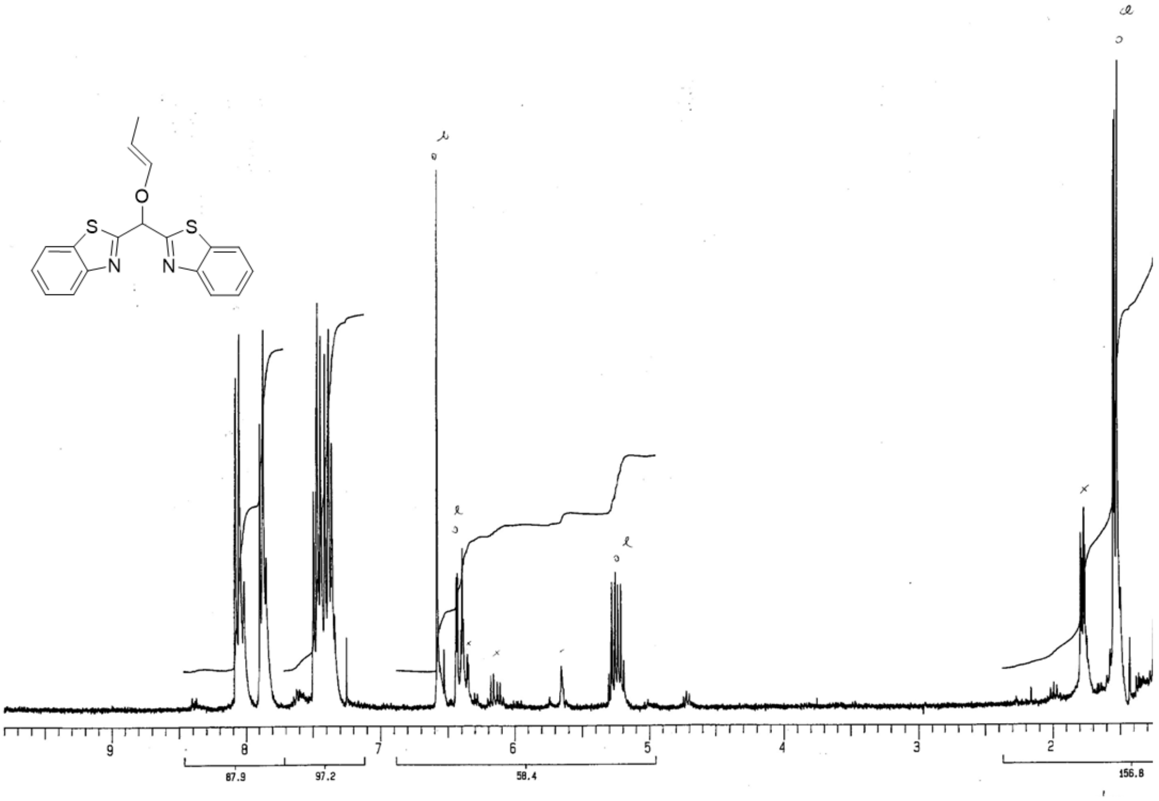

Figure 23. ^{1}H NMR spectrum ($CDCl_3$) of **4f**.

checkCIF/PLATON report

You have not supplied any structure factors. As a result the full set of tests cannot be run.

THIS REPORT IS FOR GUIDANCE ONLY. IF USED AS PART OF A REVIEW PROCEDURE FOR PUBLICATION, IT SHOULD NOT REPLACE THE EXPERTISE OF AN EXPERIENCED CRYSTALLOGRAPHIC REFEREE.

No syntax errors found. CIF dictionary Interpreting this report

Datablock: shelx

```
Bond precision:  C-C = 0.0030 A          Wavelength=0.71073

Cell:            a=18.3273(12)     b=16.2782(11)     c=4.2718(3)
                 alpha=90          beta=90           gamma=90
Temperature:     273 K

                Calculated                  Reported
Volume          1274.43(15)                 1274.43(15)
Space group     P n a 21                    P n a 21
Hall group      P 2c -2n                    P 2c -2n
Moiety formula  C15 H8 N2 O S2              ?
Sum formula     C15 H8 N2 O S2              C15 H8 N2 O S2
Mr              296.35                      296.35
Dx,g cm-3       1.545                       1.545
Z               4                           4
Mu (mm-1)       0.412                       0.412
F000            608.0                       608.0
F000'           609.19
h,k,lmax        24,21,5                     24,20,5
Nref            3264[ 1844]                 3045
Tmin,Tmax       0.929,0.940                 0.902,0.987
Tmin'           0.891

Correction method= # Reported T Limits: Tmin=0.902 Tmax=0.987
AbsCorr = MULTI-SCAN

Data completeness= 1.65/0.93       Theta(max)= 28.616

R(reflections)= 0.0255( 2843)      wR2(reflections)= 0.0691( 3045)

S = 1.076                 Npar= 181
```

The following ALERTS were generated. Each ALERT has the format
test-name_ALERT_alert-type_alert-level.
Click on the hyperlinks for more details of the test.

Alert level G

```
PLAT199_ALERT_1_G Reported _cell_measurement_temperature ..... (K)        273 Check
PLAT200_ALERT_1_G Reported   _diffrn_ambient_temperature ..... (K)        273 Check
```

```
   0 ALERT level A = Most likely a serious problem - resolve or explain
   0 ALERT level B = A potentially serious problem, consider carefully
   0 ALERT level C = Check. Ensure it is not caused by an omission or oversight
   2 ALERT level G = General information/check it is not something unexpected

   2 ALERT type 1 CIF construction/syntax error, inconsistent or missing data
   0 ALERT type 2 Indicator that the structure model may be wrong or deficient
   0 ALERT type 3 Indicator that the structure quality may be low
   0 ALERT type 4 Improvement, methodology, query or suggestion
   0 ALERT type 5 Informative message, check
```

It is advisable to attempt to resolve as many as possible of the alerts in all categories. Often the minor alerts point to easily fixed oversights, errors and omissions in your CIF or refinement strategy, so attention to these fine details can be worthwhile. In order to resolve some of the more serious problems it may be necessary to carry out additional measurements or structure refinements. However, the purpose of your study may justify the reported deviations and the more serious of these should normally be commented upon in the discussion or experimental section of a paper or in the "special_details" fields of the CIF. checkCIF was carefully designed to identify outliers and unusual parameters, but every test has its limitations and alerts that are not important in a particular case may appear. Conversely, the absence of alerts does not guarantee there are no aspects of the results needing attention. It is up to the individual to critically assess their own results and, if necessary, seek expert advice.

Publication of your CIF in IUCr journals

A basic structural check has been run on your CIF. These basic checks will be run on all CIFs submitted for publication in IUCr journals (*Acta Crystallographica*, *Journal of Applied Crystallography*, *Journal of Synchrotron Radiation*); however, if you intend to submit to *Acta Crystallographica Section C* or *E* or *IUCrData*, you should make sure that full publication checks are run on the final version of your CIF prior to submission.

Publication of your CIF in other journals

Please refer to the *Notes for Authors* of the relevant journal for any special instructions relating to CIF submission.

PLATON version of 09/11/2017; check.def file version of 08/11/2017

Datablock shelx - ellipsoid plot

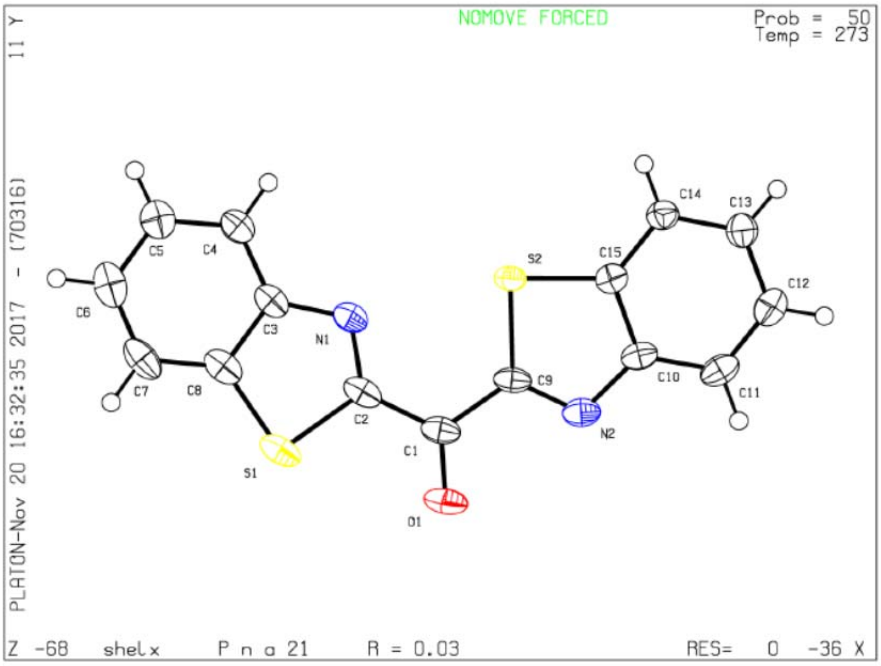

checkCIF/PLATON report

You have not supplied any structure factors. As a result the full set of tests cannot be run.

THIS REPORT IS FOR GUIDANCE ONLY. IF USED AS PART OF A REVIEW PROCEDURE FOR PUBLICATION, IT SHOULD NOT REPLACE THE EXPERTISE OF AN EXPERIENCED CRYSTALLOGRAPHIC REFEREE.

No syntax errors found. CIF dictionary Interpreting this report

Datablock: shelx

```
Bond precision:   C-C = 0.0040 A          Wavelength=0.71073

Cell:             a=3.7993(3)    b=29.361(2)        c=13.7502(10)
                  alpha=90       beta=91.769(1)     gamma=90
Temperature:      273 K

                 Calculated                   Reported
Volume           1533.1(2)                    1533.1(2)
Space group      C c                          C c
Hall group       C -2yc                       C -2yc
Moiety formula   C7 H4 N2 O S2                ?
Sum formula      C7 H4 N2 O S2                C7 H4 N2 O S2
Mr               196.24                       196.24
Dx,g cm-3        1.701                        1.700
Z                8                            8
Mu (mm-1)        0.636                        0.636
F000             800.0                        800.0
F000'            802.22
h,k,lmax         5,38,18                      4,38,18
Nref             3939[ 1979]                  3461
Tmin,Tmax        0.892,0.927                  0.912,0.958
Tmin'            0.881

Correction method= # Reported T Limits: Tmin=0.912 Tmax=0.958
AbsCorr = MULTI-SCAN

Data completeness= 1.75/0.88       Theta(max)= 28.660

R(reflections)= 0.0239( 3310)      wR2(reflections)= 0.0597( 3461)

S = 1.037                 Npar= 217
```

The following ALERTS were generated. Each ALERT has the format
      test-name_ALERT_alert-type_alert-level.
Click on the hyperlinks for more details of the test.

Alert level G

```
PLAT199_ALERT_1_G Reported _cell_measurement_temperature ..... (K)        273 Check
PLAT200_ALERT_1_G Reported   _diffrn_ambient_temperature ..... (K)        273 Check
```

```
  0 ALERT level A = Most likely a serious problem - resolve or explain
  0 ALERT level B = A potentially serious problem, consider carefully
  0 ALERT level C = Check. Ensure it is not caused by an omission or oversight
  2 ALERT level G = General information/check it is not something unexpected

  2 ALERT type 1 CIF construction/syntax error, inconsistent or missing data
  0 ALERT type 2 Indicator that the structure model may be wrong or deficient
  0 ALERT type 3 Indicator that the structure quality may be low
  0 ALERT type 4 Improvement, methodology, query or suggestion
  0 ALERT type 5 Informative message, check
```

It is advisable to attempt to resolve as many as possible of the alerts in all categories. Often the minor alerts point to easily fixed oversights, errors and omissions in your CIF or refinement strategy, so attention to these fine details can be worthwhile. In order to resolve some of the more serious problems it may be necessary to carry out additional measurements or structure refinements. However, the purpose of your study may justify the reported deviations and the more serious of these should normally be commented upon in the discussion or experimental section of a paper or in the "special_details" fields of the CIF. checkCIF was carefully designed to identify outliers and unusual parameters, but every test has its limitations and alerts that are not important in a particular case may appear. Conversely, the absence of alerts does not guarantee there are no aspects of the results needing attention. It is up to the individual to critically assess their own results and, if necessary, seek expert advice.

Publication of your CIF in IUCr journals

A basic structural check has been run on your CIF. These basic checks will be run on all CIFs submitted for publication in IUCr journals (*Acta Crystallographica*, *Journal of Applied Crystallography*, *Journal of Synchrotron Radiation*); however, if you intend to submit to *Acta Crystallographica Section C* or *E* or *IUCrData*, you should make sure that full publication checks are run on the final version of your CIF prior to submission.

Publication of your CIF in other journals

Please refer to the *Notes for Authors* of the relevant journal for any special instructions relating to CIF submission.

PLATON version of 09/11/2017; check.def file version of 08/11/2017

Datablock shelx - ellipsoid plot

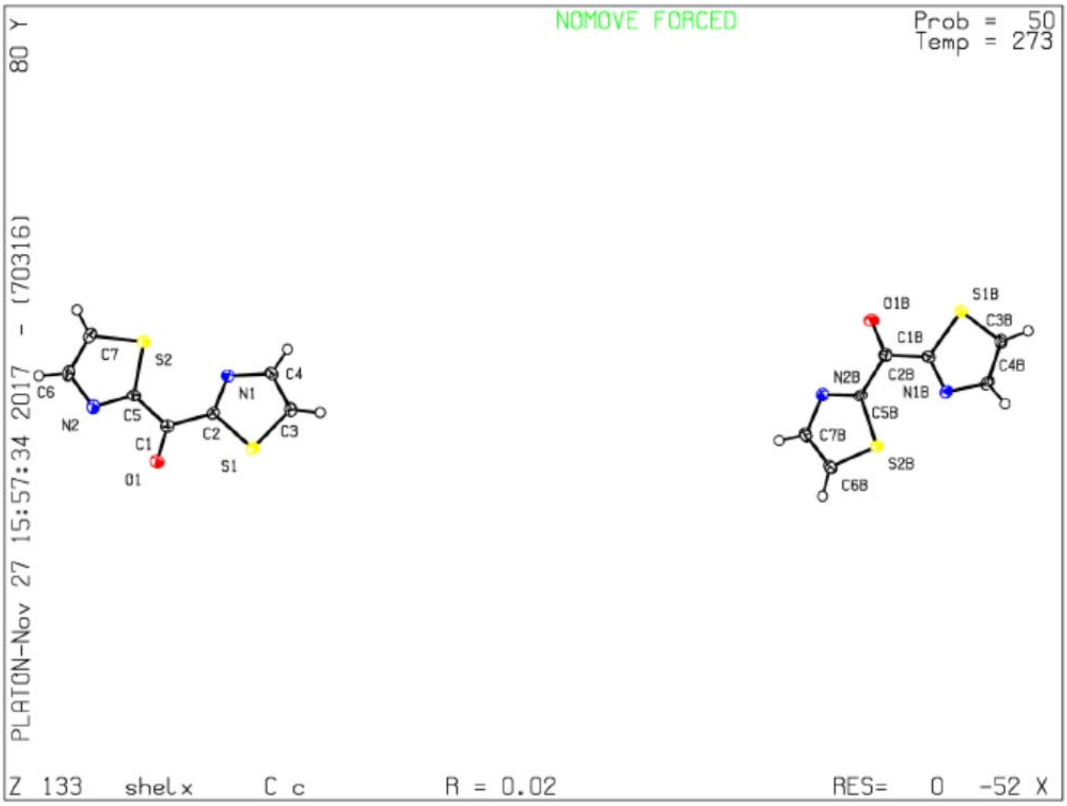

checkCIF/PLATON report

You have not supplied any structure factors. As a result the full set of tests cannot be run.

THIS REPORT IS FOR GUIDANCE ONLY. IF USED AS PART OF A REVIEW PROCEDURE FOR PUBLICATION, IT SHOULD NOT REPLACE THE EXPERTISE OF AN EXPERIENCED CRYSTALLOGRAPHIC REFEREE.

No syntax errors found. CIF dictionary Interpreting this report

Datablock: shelx

```
Bond precision:  C-C = 0.0019 A          Wavelength=0.71073

Cell:            a=13.1814(14)     b=11.9694(13)     c=7.6380(8)
                 alpha=90          beta=100.217(2)   gamma=90
Temperature:     273 K

                 Calculated                 Reported
Volume           1186.0(2)                  1186.0(2)
Space group      P 21/c                     P 21/c
Hall group       -P 2ybc                    -P 2ybc
Moiety formula   C15 H8 N2 O3               ?
Sum formula      C15 H8 N2 O3               C15 H8 N2 O3
Mr               264.23                     264.23
Dx,g cm-3        1.480                      1.480
Z                4                          4
Mu (mm-1)        0.106                      0.106
F000             544.0                      544.0
F000'            544.27
h,k,lmax         17,15,10                   16,15,10
Nref             2857                       2785
Tmin,Tmax        0.964,0.979                0.890,0.948
Tmin'            0.964

Correction method= # Reported T Limits: Tmin=0.890 Tmax=0.948
AbsCorr = MULTI-SCAN

Data completeness= 0.975          Theta(max)= 27.989

R(reflections)= 0.0348( 2113)     wR2(reflections)= 0.0947( 2785)

S = 0.993               Npar= 182
```

The following ALERTS were generated. Each ALERT has the format
test-name_ALERT_alert-type_alert-level.
Click on the hyperlinks for more details of the test.

```
Alert level B
PLAT230_ALERT_2_B Hirshfeld Test Diff for  N1        --C2        ..        9.6 s.u.
PLAT230_ALERT_2_B Hirshfeld Test Diff for  N2        --C9        ..        7.4 s.u.
```

```
Alert level G
PLAT199_ALERT_1_G Reported _cell_measurement_temperature ..... (K)        273 Check
PLAT200_ALERT_1_G Reported  _diffrn_ambient_temperature ..... (K)        273 Check
PLAT398_ALERT_2_G Deviating  C-O-C    Angle From 120 for O2             103.4 Degree
PLAT398_ALERT_2_G Deviating  C-O-C    Angle From 120 for O3             103.6 Degree
```

```
   0 ALERT level A = Most likely a serious problem - resolve or explain
   2 ALERT level B = A potentially serious problem, consider carefully
   0 ALERT level C = Check. Ensure it is not caused by an omission or oversight
   4 ALERT level G = General information/check it is not something unexpected

   2 ALERT type 1 CIF construction/syntax error, inconsistent or missing data
   4 ALERT type 2 Indicator that the structure model may be wrong or deficient
   0 ALERT type 3 Indicator that the structure quality may be low
   0 ALERT type 4 Improvement, methodology, query or suggestion
   0 ALERT type 5 Informative message, check
```

It is advisable to attempt to resolve as many as possible of the alerts in all categories. Often the minor alerts point to easily fixed oversights, errors and omissions in your CIF or refinement strategy, so attention to these fine details can be worthwhile. In order to resolve some of the more serious problems it may be necessary to carry out additional measurements or structure refinements. However, the purpose of your study may justify the reported deviations and the more serious of these should normally be commented upon in the discussion or experimental section of a paper or in the "special_details" fields of the CIF. checkCIF was carefully designed to identify outliers and unusual parameters, but every test has its limitations and alerts that are not important in a particular case may appear. Conversely, the absence of alerts does not guarantee there are no aspects of the results needing attention. It is up to the individual to critically assess their own results and, if necessary, seek expert advice.

Publication of your CIF in IUCr journals

A basic structural check has been run on your CIF. These basic checks will be run on all CIFs submitted for publication in IUCr journals (*Acta Crystallographica*, *Journal of Applied Crystallography*, *Journal of Synchrotron Radiation*); however, if you intend to submit to *Acta Crystallographica Section C* or *E* or *IUCrData*, you should make sure that full publication checks are run on the final version of your CIF prior to submission.

Publication of your CIF in other journals

Please refer to the *Notes for Authors* of the relevant journal for any special instructions relating to CIF submission.

PLATON version of 09/11/2017; check.def file version of 08/11/2017

Datablock shelx - ellipsoid plot

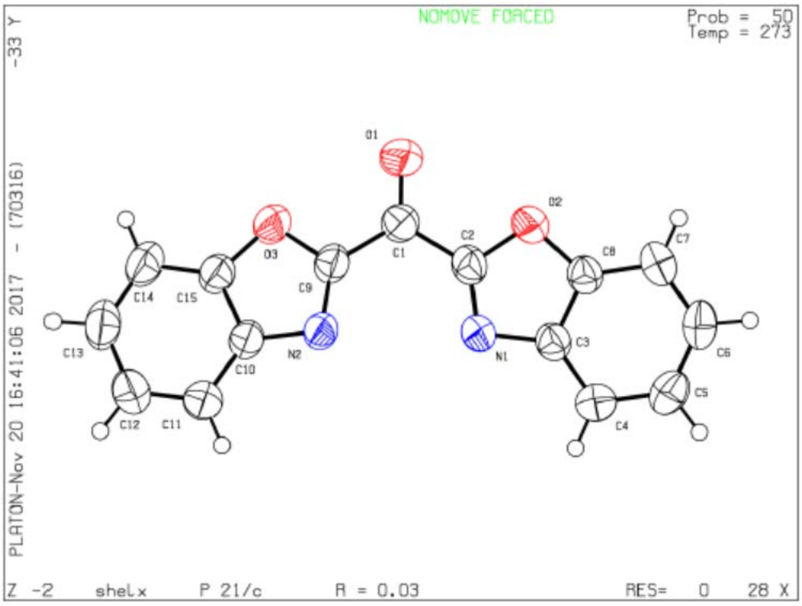

checkCIF/PLATON report

You have not supplied any structure factors. As a result the full set of tests cannot be run.

THIS REPORT IS FOR GUIDANCE ONLY. IF USED AS PART OF A REVIEW PROCEDURE FOR PUBLICATION, IT SHOULD NOT REPLACE THE EXPERTISE OF AN EXPERIENCED CRYSTALLOGRAPHIC REFEREE.

No syntax errors found.   CIF dictionary   Interpreting this report

Datablock: shelx

```
Bond precision:  C-C = 0.0026 A          Wavelength=0.71073

Cell:            a=7.3636(7)    b=15.5117(14)     c=14.4556(13)
                 alpha=90       beta=103.544(1)   gamma=90
Temperature:     273 K

                Calculated                  Reported
Volume          1605.2(3)                   1605.2(3)
Space group     P 21/n                      P 21/n
Hall group      -P 2yn                      -P 2yn
Moiety formula  C17 H14 N4 O, 2(H2 O)       ?
Sum formula     C17 H18 N4 O3               C17 H18 N4 O3
Mr              326.35                      326.35
Dx,g cm-3       1.350                       1.350
Z               4                           4
Mu (mm-1)       0.095                       0.095
F000            688.0                       688.0
F000'           688.30
h,k,lmax        9,19,17                     9,19,17
Nref            3239                        3230
Tmin,Tmax       0.983,0.991                 0.876,0.945
Tmin'           0.981

Correction method= # Reported T Limits: Tmin=0.876 Tmax=0.945
AbsCorr = MULTI-SCAN

Data completeness= 0.997            Theta(max)= 26.237

R(reflections)= 0.0448( 2291)       wR2(reflections)= 0.1366( 3230)

S = 1.090                 Npar= 231
```

The following ALERTS were generated. Each ALERT has the format
test-name_ALERT_alert-type_alert-level.
Click on the hyperlinks for more details of the test.

```
Alert level B
PLAT417_ALERT_2_B Short Inter D-H..H-D        H2W      ..H3W        ..        1.93 Ang.
PLAT420_ALERT_2_B D-H Without Acceptor         O2W      --H3W        .       Please Check

Alert level C
PLAT355_ALERT_3_C Long   O-H (X0.82,N0.98A)  O2W      - H3W        .         1.04 Ang.

Alert level G
PLAT002_ALERT_2_G Number of Distance or Angle Restraints on AtSite          6 Note
PLAT172_ALERT_4_G The CIF-Embedded .res File Contains DFIX Records          1 Report
PLAT199_ALERT_1_G Reported _cell_measurement_temperature ..... (K)        273 Check
PLAT200_ALERT_1_G Reported   _diffrn_ambient_temperature ..... (K)        273 Check
PLAT860_ALERT_3_G Number of Least-Squares Restraints .............          4 Note

   0 ALERT level A = Most likely a serious problem - resolve or explain
   2 ALERT level B = A potentially serious problem, consider carefully
   1 ALERT level C = Check. Ensure it is not caused by an omission or oversight
   5 ALERT level G = General information/check it is not something unexpected

   2 ALERT type 1 CIF construction/syntax error, inconsistent or missing data
   3 ALERT type 2 Indicator that the structure model may be wrong or deficient
   2 ALERT type 3 Indicator that the structure quality may be low
   1 ALERT type 4 Improvement, methodology, query or suggestion
   0 ALERT type 5 Informative message, check
```

It is advisable to attempt to resolve as many as possible of the alerts in all categories. Often the minor alerts point to easily fixed oversights, errors and omissions in your CIF or refinement strategy, so attention to these fine details can be worthwhile. In order to resolve some of the more serious problems it may be necessary to carry out additional measurements or structure refinements. However, the purpose of your study may justify the reported deviations and the more serious of these should normally be commented upon in the discussion or experimental section of a paper or in the "special_details" fields of the CIF. checkCIF was carefully designed to identify outliers and unusual parameters, but every test has its limitations and alerts that are not important in a particular case may appear. Conversely, the absence of alerts does not guarantee there are no aspects of the results needing attention. It is up to the individual to critically assess their own results and, if necessary, seek expert advice.

Publication of your CIF in IUCr journals

A basic structural check has been run on your CIF. These basic checks will be run on all CIFs submitted for publication in IUCr journals (*Acta Crystallographica*, *Journal of Applied Crystallography*, *Journal of Synchrotron Radiation*); however, if you intend to submit to *Acta Crystallographica Section C* or *E* or *IUCrData*, you should make sure that full publication checks are run on the final version of your CIF prior to submission.

Publication of your CIF in other journals

Please refer to the *Notes for Authors* of the relevant journal for any special instructions relating to CIF submission.

PLATON version of 09/11/2017; check.def file version of 08/11/2017

Datablock shelx - ellipsoid plot

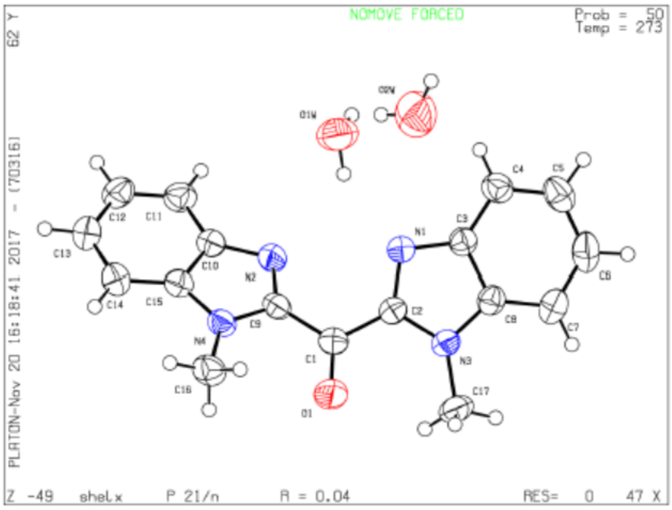

Supplement: Supplementary file 1 [file molecules-23-00171-s001.pdf]
